# Cholecalciferol decreases inflammation and improves vitamin D regulatory enzymes in lymphocytes in the uremic environment: A randomized controlled pilot trial

**DOI:** 10.1371/journal.pone.0179540

**Published:** 2017-06-30

**Authors:** José Tarcisio G. Carvalho, Marion Schneider, Lilian Cuppari, Caren C. Grabulosa, Danilo T. Aoike, Beata Marie Q. Redublo, Marcelo C. Batista, Miguel Cendoroglo, Rosa Maria Moyses, Maria Aparecida Dalboni

**Affiliations:** 1 Division of Nephrology- Universidade Federal São Paulo, UNIFESP, São Paulo, São Paulo, Brazil; 2 Post-graduate Program in Medicine, Universidade Nove de Julho/UNINOVE, São Paulo, São Paulo, Brazil; 3 Hospital Israelita Albert Einstein, São Paulo, São Paulo, Brazil; Medizinische Universitat Graz, AUSTRIA

## Abstract

It has been reported that vitamin D regulates the immune system. However, whether vitamin D repletion modulates inflammatory responses in lymphocytes from dialysis patients is unclear. In the clinical trial, thirty-two (32) dialysis patients with 25 vitamin D ≤ 20ng/mL were randomized to receive either supplementation of cholecalciferol 100,000 UI/week/3 months (16 patients) or placebo (16 patients). In the *in vitro* study, B and T lymphocytes from 12 healthy volunteers (HV) were incubated with or without uremic serum in the presence or absence of 25 or 1,25 vitamin D. We evaluated the intracellular expression of IL-6, IFN-γ TLR7, TLR9, VDR, CYP27b1 and CYP24a1 by flow cytometry. We observed a reduction in the expression of TLR7, TLR9, INF-γ and CYP24a1 and an increase in VDR and CYP27b1 expression in patients which were supplemented with cholecalciferol, whereas no differences were found in the placebo group. Uremic serum increased the intracellular expression of IL-6, IFN-γ, TLR7, TLR9, VDR, CYP27b1 and CYP24a1. Treatment with 25 or 1,25 vitamin D decreased IL-6 and TLR9. CYP24a1 silencing plus treatment with 25 and/or 1,25 vitamin D had an additional reduction effect on IL-6, IFN-γ, TLR7 and TLR9 expression. This is the first study showing that cholecalciferol repletion has an anti-inflammatory effect and improves vitamin D intracellular regulatory enzymes on lymphocytes from dialysis patients.

## Introduction

Dysregulation of immune and inflammatory responses in uremia, specially related to lymphocyte activation is involved in the pathophysiology of cardiovascular (CV) and infectious diseases, the most common causes of mortality and morbidity among patients with chronic kidney disease (CKD) [1–3].

CKD patients exhibit low levels of 25(OH)D_3_ and 1,25(OH)_2_D_3_ (25 and 1,25 vitamin D), mainly when glomerular filtration rate is lower than 60 mL/min/1,73 m^2^, and in dialysis patients (DP) [4, 5]. In recent years, both 25 and 1,25 vitamin D have been described to have an important role in the regulation of immune functions, apart from calcium and bone homeostasis [4–6].

Vitamin D (cholecalciferol) is mainly synthesized in the skin when a cholesterol precursor, 7-dehydroxycholesterol, is exposed to ultraviolet light [7, 8]. Activation occurs when the substance undergoes 25-hydroxylation in the liver and 1-hydroxylation by renal 1-α hydroxylase (CYP27b1) that convert 25 to 1,25 vitamin D (an active form of biological activity) in the kidney, and the excess of 25 and 1,25 vitamin D is degraded in inactive form by renal 24-hydroxylase (CYP24a1) [9, 10]. Local 1,25 vitamin D promotes up-regulation of renal CYP24 activity. The induction of CYP24, and catabolism of 1,25 vitamin D by itself is an important feedback loop which avoids 1,25 vitamin D toxicity in target tissues [9, 10]. Metabolisms of active vitamin D are also regulated by Parathyroid hormone (PTH) and Fibroblast growth factor 23 (FGF-23) [9–11]. PTH secreted by the parathyroid glands promotes absorption of calcium from the bone, and in the kidney proximal tubular cells acts to increase renal calcium resorption and phosphate excretion, and also acts to stimulate 1-α hydroxylase (CYP27b1) production to convert 25 to 1,25 vitamin D [9–13]. FGF-23 a phosphaturic hormone produced by osteoblasts/osteocytes is also one of the main regulators of 1,25 vitamin D metabolism, which downregulates the 1,25 vitamin D by diminish CYP27b1 and increase CYP24a1 production [9, 11, 13]. Lower levels of 25 and 1,25 vitamin D have been associated with elevated serum level of Fibroblast growth factor 23 (FGF-23) and decreased kidney CYP27b1 due to kidney disease progress [13, 14]. Decreased 1,25 vitamin D levels may be due in part to decreased kidney CYP27b1, as a consequence of kidney function degeneration. However, loss of CYP27b1 cannot account for the concomitant loss of 25 vitamin D. Another possible explanation is that elevated vitamin D catabolism by CYP24a1 [14].

In the last years, it has been reported the production of 1,25 vitamin D occurs not only in the kidneys, but also in extrarenal sites [6, 15], and others cell types also express vitamin D receptor (VDR) and regulatory enzymes, such as CYP27b1 and CYP24a1. The extrarenal synthesis of vitamin D might be linked to autocrine and paracrine biologic regulatory mechanisms, mainly in cells of the immunologic system [6, 15].

In fact, an important role of 25 and 1,25 vitamin D in the immune system involves the control of inflammatory responses. Recently, some studies investigated the impact of supplementation with cholecalciferol on systemic inflammation in CKD patients. Stubbs *et al* (2010) observed a reduction in serum TNF-α, IL-6 and CRP in hemodialysis patients after treatment with cholecalciferol [16]. In addition, Alvarez *et al* (2014) observed a decrease in Monocyte Chemoattractant Protein-1 (MCP-1) expression in patients in pre–dialysis stages 2 and 3 which received cholecalciferol supplementation [17].

Toll-like receptors (TLR) represent an important pathway in the signalling of inflammatory responses. They recognize highly conserved structural motifs known as pathogen-associated microbial patterns (PAMPs), which are exclusively expressed by microbial pathogens, or danger-associated molecular patterns (DAMPs), which are endogenous molecules or non-pathogens products [18–20]. In turn, activation of TLR by ligands leads to a production of pro-inflammatory cytokines, via signaling pathways activating transcription factors, such as the nuclear factor kappa B (NF-κB) [18, 19].

The impact of renal dysfunction on TLR expression is poorly understood. Some studies have shown that patients on dialysis have a low TLR4 expression on monocytes, resulting in a decreased ability to recognize and synthesize cytokines in response to a bacterial challenge [21–23]. In contrast, other studies report that monocytes from CKD patients exhibit high expression of TLRs (TLR2 and TLR4) as well as a high production of pro-inflammatory cytokines such as TNF-α and IL-6 [24]. B and T lymphocytes also express TLRs, such as TLR7 and TLR9 [24, 25]. These TLRs are intracellular pattern-recognition receptors that recognize specific pathogen ligands, especially viral double-stranded RNA as influenza and hepatitis virus. Once these receptors are activated, a limited production of inflammatory cytokines occurs, as a physiologic response against these pathogens [19]. However, there are few studies investigating the mechanisms of inflammatory responses on lymphocytes from CKD patients [2, 25, 26]. Moreover, there is no report that had investigated TLRs expression, VDR and vitamin D regulatory enzymes in these cells. It is also unclear whether vitamin D could modulate these receptors in a uremic environment.

Therefore, the purpose of this study was to evaluate the effect of cholecalciferol supplementation on the expression of TLR7, TLR9, IFN-γ, IL-6, VDR, CYP27b1 and CYP24a1 in B and T lymphocytes from dialysis patients with hypovitaminosis D. *In vitro*, we assessed the effect of 25 and 1,25 vitamin D on lymphocytes from healthy volunteers (HV) in the presence or absence of uremic serum related to inflammatory response mechanisms, intracellular VDR and vitamin D regulatory enzymes.

## Material and methods

### Clinical trial

#### Subjects

**Fig 1:**
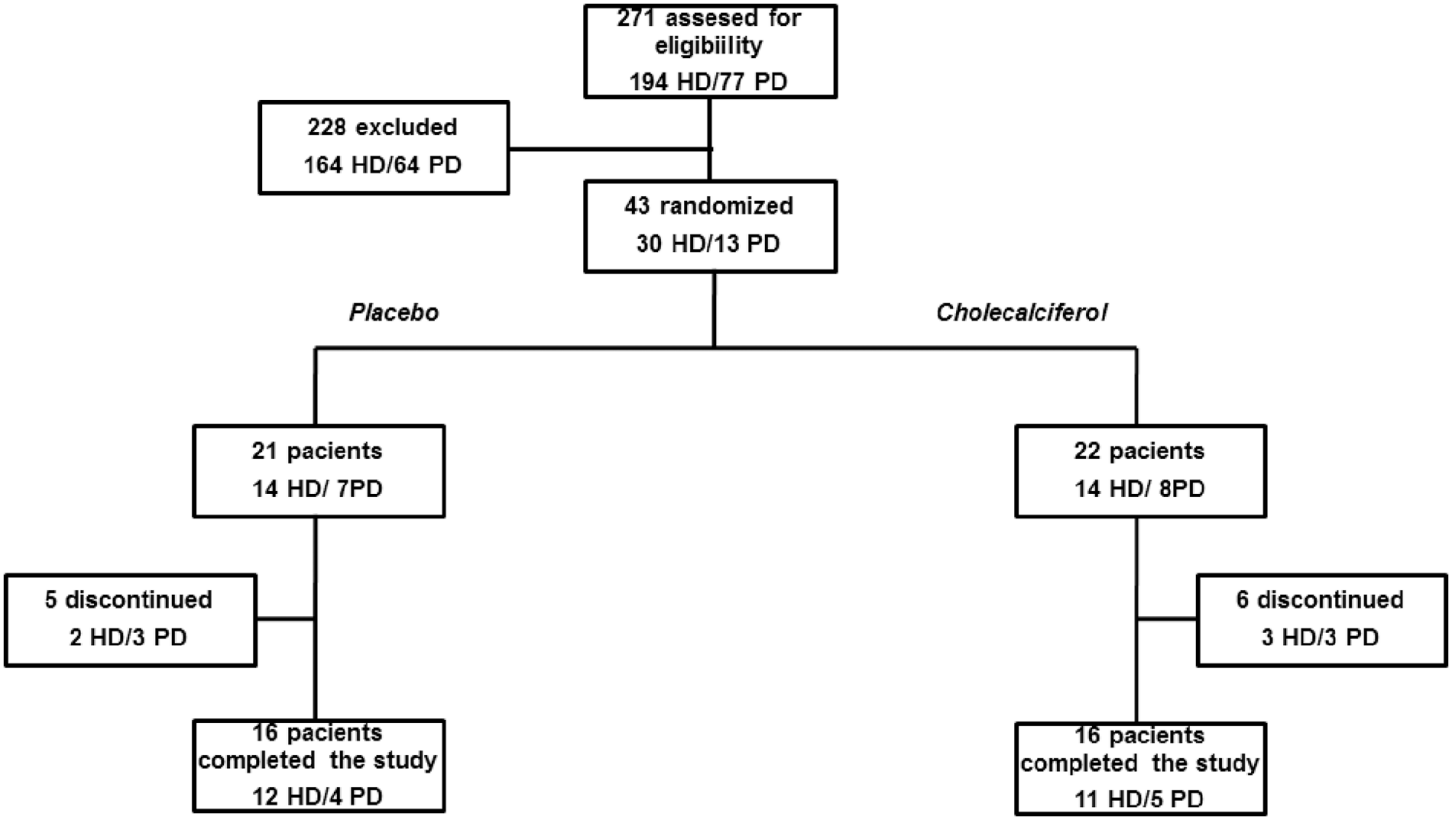
Flowchart of participants through the study.

As shown in Fig 1, from September 2012 to May 2014, 271 patients were screened (194, Hemodialysis-HD and 77, peritoneal dialysis-PD). Among these, 92 patients were excluded due to the use of vitamin D compounds, 52 were excluded because the serum 25 vitamin D was greater than 20 ng/mL, and 84 patients did not meet other inclusion criteria. Therefore, 43 patients were randomized. However, eleven patients (n = 11) were lost during follow-up due to hospitalization (n = 6), death (n = 1) or lack of compliance with the study protocol (n = 4). Therefore, the present study was completed with a total of 32 patients (23 HD/9 DP). HD patients underwent on conventional HD for 4 h, 3 times a week, using bicarbonate-buffered dialysate and polysulfone dialyzer membranes. Most of the PD patients (87%) were treated by automated PD using a glucose-based solution. The study was approved by the Human Investigation Review Committee of the Federal University of São Paulo, and all participants provided written informed consent for study *in vivo* and *in vitro*. The trial is registered at ClinicalTrials.gov #NCT01974245.

These patients were a part used for the already published article by our group [27]. In brief; previously 348 patients (194HD/154PD) were screened, from which 55 patients (30HD/25PD) were randomized. Among these, 38 patients (23 HD/15 DP) completed the protocol of the study.

In the present study dialysis patients from single dialysis unit of the Oswaldo Ramos Foundation (São Paulo, Brazil), aged between 18 and 80 years were included in this study. Inclusion criteria were dialysis vintage of at least 3 months and 25 vitamin D serum levels < 20 ng/mL. The exclusion criteria were use of any vitamin D compound, glucocorticoids or immunosuppressant drugs, history of liver failure, intestinal malabsorption, malignancy, autoimmune disease, active infection, HIV, peritonitis in the last month or elevated serum ionized calcium (>1.40 mmol/L).

#### Protocol

The present study was a 12-week randomized, double-blind, placebo-controlled clinical trial. Patients were randomized to receive either cholecalciferol or placebo. Participants and researchers were blinded. The patients were assigned to cholecalciferol or control groups after a blocked randomization procedure by using a random block of 4 participants. An independent researcher generated a computerized random list and the allocation sequence was concealed in a closed box. A researcher was responsible for label and numbers all containers according to the random schedule.

Patients in the cholecalciferol group received 50,000 IU of cholecalciferol (1,000 IU/drop; Magister Pharmacy, São Paulo, SP, Brazil) twice a week, while the control group received a placebo solution. Pharmaceutical presentation of the placebo and cholecalciferol were identical. To minimize the effects of vitamin D synthesis by the skin, all patients received sunscreen (SPF 30) and were instructed to wear it during the study period. Medication adherence was checked through pill count. Patients were encouraged to follow the protocol during the monthly visits and through phone calls. Venous blood was drawn at baseline and after 3 months, before starting the first hemodialysis session of the week.

#### Detection of serum levels of 25 vitamin D, parathyroid hormone (PTH), fibroblast growth factor 23 (FGF-23), calcium and phosphorus

10 mL of blood samples were collected after eight hours of fasting from all participants at baseline and 12 weeks after supplementation with cholecalciferol. To measure serum biochemical parameters, 8 mL from each blood sample was left for 30 minutes in the dark at 8°C to cloth and after that, it was centrifuged at 500 g, 8°C for 10 minutes. 25 vitamin D measurement was performed by chemiluminescence immunoassay (Abbott, Wiesbaden, Germany); PTH 1–84 was measured by immunoradiometric assay (DiaSorin, Rome, Italy) and FGF-23 was measured by ELISA (R&D Systems, Minneapolis, USA) according to the manufacturer’s instructions. Respective inter- and intra-assay coefficients of variation from FGF-23 were 4.1% and 5.9%. Serum calcium and phosphorus were measured by Roche Cobas Integra auto analyzer (F Hoffmann-La Roche Ltd, Basel, Switzerland).

#### Measurement of TLR7, TLR9, IFN-γ, IL-6, VDR, CYP27b1 and CYP24a1

An aliquot of 100 μL of heparinized whole blood each patient was incubated with 10 μL of PerCP-labeled CD3 antibody and 5uL of PE-CY7-labeled CD19 antibody (BD Biosciences, San Diego, USA) in polypropylene tubes for 15 min in the dark at room temperature to characterize T and B lymphocytes, respectively. Red blood cells were lysed with FACS lysing solution (BD Biosciences, San Diego, USA) for 10 min. After washing, cells were permeabilized with FACS permeabilizing solution (BD Biosciences, San Diego, USA) for 20 min. Then, cells were stained with VDR, CYP27B1, CYP24 antibodies (Santa Cruz, San Diego, USA). All primary antibodies were added at a concentration of 1 μg/mL and were incubated for 30 min at 8°C. After washing 3 times with PBS, cells were incubated with 3μg/mL of respective secondary antibodies (APC-conjugated anti-human VDR; Alexa-Fluor 647-conjugated anti-human CYP27B1 and Alexa-Fluor 488-conjugated anti-human CYP24) for 15 min at 8°C.

In another tube, 100 μL of heparinized whole blood from each patient was pre-treated with monensin (BD Biosciences, San Diego, USA) and incubated with CD3 and CD19 antibody. Monensin is a protein transport inhibitor which blocks the extravasation of intracellular cytokines necessary for staining protocol by flow cytometry. After lysing, cells were permeabilized as described above to intracellular stain with 5 μL pf FITC-labeled IL-6 (BD Biosciences, San Diego, USA), 10 μL of PE-labeled TLR7 antibody (R&D Systems, Mineapolis, USA), 5 μL of APC-Cy7-labeled IFN-γ (eBiosciences, San Diego, USA) and 10 μL of APC-labeled TLR9 (BD Biosciences, San Diego, USA) antibodies. All monoclonal are incubated at 8°C for 30 minutes to intracellular stain.

The detection of all antibodies was performed by a flow cytometer (FacsCanto I, BD Biosciences, San Diego, USA). For each sample, 30.000 cells were acquired and analyzed. Forward scatter and side scatter were used to gate lymphocytes and to exclude cellular debris. The gated data of CD3+ (T lymphocytes), CD19+ (B lymphocytes) and the expression of TLR7, TLR9, IFN-γ, IL-6, VDR, CYP27B1 and CYP24 were presented as mean fluorescence intensity peak (MFI). The Fluorescence Minus One Control method (FMO control) was used to identify and to establish gate cells and to control overlapping fluorophores (S1 Fig).

### *In vitro* assays

#### Serum pool from healthy volunteers and uremic patients

We used pooled serum from 20 healthy volunteers recruited from the medical staff; the blood samples were collected after eight hours of fasting from each healthy volunteer. For the hemodialysis patient, we pooled serum of 30 hemodialysis patients after eight hours of fasting and before starting the first hemodialysis session of the week. We performed detection of biochemical characteristics of healthy and uremic serum pool, respectively: creatinine (0.82 *versus* 8.92 mg/dL); PTH (23 *vs* 392 pg/mL); calcium (9.7 *vs* 7.4 mg/dL); phosphate (3.4 *vs* 4.3 mg/dL) and 25 vitamin D (19 *vs* 10 ng/mL). PTH, calcium, phosphate, 25 vitamin D were measured as described previously. Creatinine was measured by kinetic Jaffe colorimetric method.

#### Human peripheral blood mononuclear cells (PBMC) isolation

Human peripheral blood mononuclear cells (PBMC) were isolated from heparinized venous blood of 12 healthy donors by Ficoll-Hypaque density gradient centrifugation (Sigma-Aldrich, St. Louis, USA). PBMCs (5×10^6^) were cultured in RPMI-1640 and antibiotics (100 U/ml penicillin and 100 μg/ml streptomycin) in the presence or absence of 25 vitamin D (30ng/mL) or 1,25 vitamin D (0.5ng/mL) (Sigma-Aldrich, St. Louis, USA) with healthy serum pool or uremic serum pool (50%) for 24 h at 37°C with 5% CO_2_. Following the incubation, monensin was added in cell culture.

The conditions of incubations were: **1)** PBMC plus healthy serum pool; **2)** PBMC plus healthy serum pool plus 25 vitamin D; **3)** PBMC plus healthy serum pool plus 1,25 vitamin D; **4)** PBMC plus uremic serum pool; **5)** PBMC plus uremic serum pool plus 25 vitamin D and **6)** PBMC plus uremic serum pool plus 1,25 vitamin D.

#### Measurement of TLR7, TLR9, IFN-γ, IL-6, VDR, CYP27b1 and CYP24a1

5 x 10^5^ of PBMC from each culture condition were incubated with CD3 and CD19 antibodies (BD Biosciences, San Diego, USA). The PBMC were permeabilized as described in the clinical trial methods, to stain the intracellular expression of VDR, CYP27b1, CYP24a1, IL-6, TLR7, IFN-γ and TLR9 and analyzed by flow cytometry.

#### siRNA-mediated CYP24a1 gene silencing

PBMC (1×10^6^) from each condition were mixed with 1 μL of Atractine (Qiagen. Hilden, Germany) solution and 2 μg of siRNA-mediated CYP24a1 gene silencing or non-silencing control siRNA (Ambion, Carlsbad, USA) according to the manufacturer’s instructions. After 24 h, the expression of TLR7, TLR9, IFN-γ, IL-6, VDR, CYP27b1 and CYP24a1 was detected by flow cytometry.

#### Effect of FGF-23 on CYP24a1 expression on PBMC

To evaluate whether FGF-23 could modulate the expression of CYP24a1 on PBMC as occur in renal cells, we added recombinant human FGF-23 antibody at the concentration of 100 ng/ml for 24 h at 37°C with 5% CO_2_ (R&D Systems, Minneapolis, USA) as follows: **1)** PBMC plus healthy serum pool; **2)** PBMC plus healthy serum pool plus FGF-23; **3)** PBMC plus uremic serum pool and **4)** PBMC plus uremic serum pool plus FGF-23). After incubation, the expression of CYP24a1 was evaluated by flow cytometry.

#### Detection of NF-κB activity on PBMC

To evaluate whether uremic serum pool, 25 and 1,25 vitamin D could modulate NF-κB, PBMC were evaluated in each condition described above for NF-κB activity, according to the manufacturer's instructions (NF-κB p50/p65 Transcription Factor Assay Kit, eBioscience, San Diego, USA). The respective inter- and intra-assay coefficients of variation were 4.1% and 5.9%.

## Statistical analysis

A normality test was performed for all variables in the *in vivo* and *in vitro* studies. Data were expressed as the mean ± standard deviation (SD) for variables with normal distribution, as the median and interquartile range for skewed distribution, and as frequencies for categorical variables. Student´s t-test or Pearson Chi-square test were used to compare the baseline characteristics of dialysis patients between the 2 groups, as appropriate. Comparisons between baseline and after 12 weeks were performed using General linear model test or Wilcoxon signed-rank test and Mann-Whitney test, as appropriate. In the *in vitro* study analysis, we used nonparametric ANOVA Kruskal-Wallis for comparisons between groups and Games-Howell post-hoc test to determine differences between groups. Correlations were done using Pearson's test. Statistical significance was defined as p<0.05. All statistical analyses were conducted using Statistical Package for Social Sciences for Windows version 18.0 (SPSS Inc., Chicago, IL, USA).

## Results

### Clinical trial

All patients were analyzed as dialysis group (DP), since we did not observe difference in the expression of TLR7, TLR9, IL-6, IFN-γ, VDR, CYP27b1 and CYP24a1 between HD and PD patients (S1 Table). At baseline, age, gender, etiology of CKD, and season of the year were not different between cholecalciferol and placebo groups (S2 Table).

As expected, there was an increase in serum 25 vitamin D and reduced levels of PTH after cholecalciferol supplementation. Ionized and total calcium and phosphorus remained unchanged in both groups. However, PTH increased only in the placebo group, whereas a significant difference in serum FGF-23 was detected by the group-time interaction analysis (Table 1).

**Table 1:** Serum levels of calcium, vitamin D, Phosphorus (P), PTH and FGF-23.

	*PLACEBO* *(n = 16)*			*CHOLECALCIFEROL* *(n = 16)*			
	*Baseline*	*12-week*	P*	*Baseline*	*12-week*	P^#^	P^†^
Ca (i) (mg/dL)	1.23±0.07	1.25±0.09	0.74	1.23±0.10	1.24±0.01	0.58	0.50
							
Ca(total)(mg/dL)	8.76±0.59	8.86±0.78	0.46	8.64±0.81	8.74±0.74	0.26	0.52
							
25(OH)D (ng/L)	13±4	15±9	0.41	16±4	43±13	**0.01**	**0.01**
							
P (mg/dL)	5.37±1.45	5.57±1.83	0.45	5.19±1.66	5.32±1.68	0.50	0.60
							
PTH (pg/mL)	260 (70–510)	330 (28–595)	**0.04**	385 (128–640)	300(85–520)	**0.03**	**0.01**
							
FGF-23 (pg/mL)	2215 (220–7720)	1870(240–6140)	0.30	1360(204–3960)	1470(180–5200)	0.40	0.01

*General Linear Model*:

P*: 12-week vs Baseline placebo group;

P^**#**^: 12-week vs Baseline cholecalciferol group.

P^**†**^: group-time interaction

As shown in Table 2, cholecalciferol therapy resulted in a significant reduction in TLR7, TLR9, IFN-γ and CYP24a1 expression and an increase in CYP27b1 and VDR expression on B and T lymphocytes compared to placebo group. However, no differences were found between the groups in respect to IL-6 expression on B or T lymphocytes.

**Table 2:** Expression of TLR7, TLR9, IL-6, IFN-γ, VDR, CYP27b1 and CYP24a1 in B and T lymphocytes.

	*PLACEBO* *(n = 16)*			*CHOLECALCIFEROL* *(n = 16)*			
	*Baseline*	*12-week*	P*	*Baseline*	*12-week*	P^#^	P^†^
*B lymphocytes*							
TLR7	296±185	259±60	0.49	305±78	252±45	**0.01**	**0.01**
TLR9	1171±585	1387±573	0.61	1864±700	1084±370	**0.001**	**0.01**
IL-6	227±105	208±59	0.48	252±92	236±83	0.50	0.83
IFN-γ	567±175	705±295	0.21	900±480	640±270	**0.03**	**0.02**
VDR	871±240	905±330	0.47	730±360	965±440	**0.05**	**0.001**
CYP27b1	296±100	284±70	0.39	245±38	442±380	**0.04**	**0.004**
CYP24a1	234±99	217±90	0.52	284±136	200±86	**0.001**	**0.009**
*T lymphocytes*							
TLR7	324±80	396±89	0.10	419±280	286±135	**0.03**	**0.01**
TLR9	1426±615	1290±405	0.12	1548±560	969±390	**0.02**	**0.004**
IL-6	222±105	229±140	0.82	276±140	252±220	0.70	0.90
IFN-γ	807±205	1031±680	0.22	908±300	646±240	**0.03**	**0.04**
VDR	800±250	820±300	0.87	626±130	840±180	**0.04**	**0.02**
CYP27b1	320±80	367±330	0.64	250±42	435±311	**0.01**	**0.03**
CYP24a1	200±63	180±53	0.66	260±122	173±65	**0.002**	**0.005**

*General Linear Model*:

P*: 12-week vs Baseline placebo group;

P^**#**^: 12-week vs Baseline cholecalciferol group.

P^**†**^: group-time interaction

We observe a negative correlation between VDR with TLR7, TLR9 and IFN-γ. We did not observe a correlation between serum levels of PTH or FGF-23 with CYP27b1 or CYP24a1 expression, respectively (S3 Table).

### In vitro

Uremic serum increased the expression of TLR7, TLR9, IL-6, IFN-γ, VDR, CYP27b1 and CYP24a1 in B and T lymphocytes compared to the effect of healthy serum. 25 and 1,25 vitamin D decreased TLR9 and IL-6 expression in B lymphocytes and TLR7 and TLR9 expression in T lymphocytes (S4 Table).

**Fig 2:**
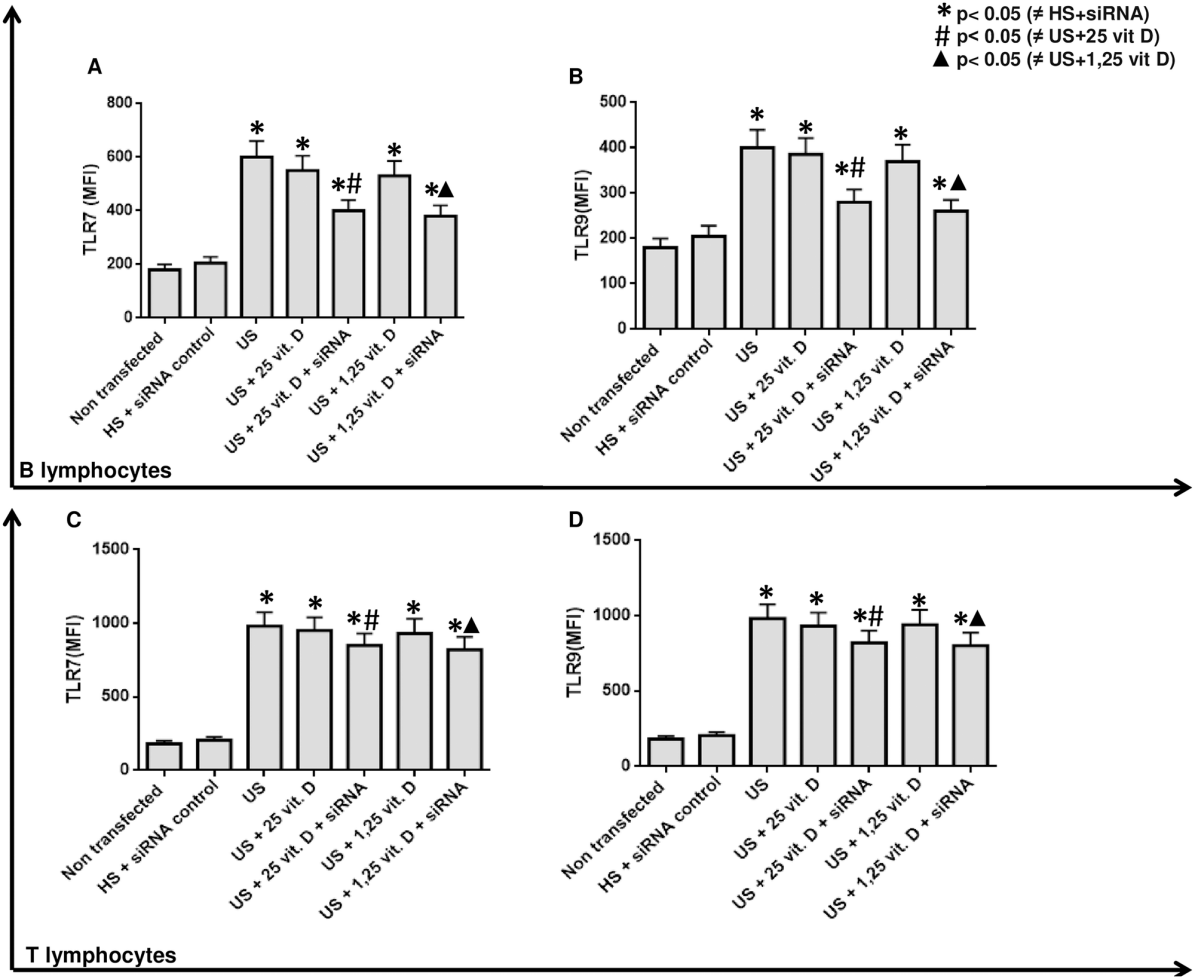
Effect of 25 and 1,25 vitamin D on TLR7 and TLR9 expression (MFI) in B lymphocytes (Figs A and B) and T lymphocytes (Figs C and D) in presence of healthy or uremic serum (US) and after CYP24 silencing (siRNA).

**Fig 3:**
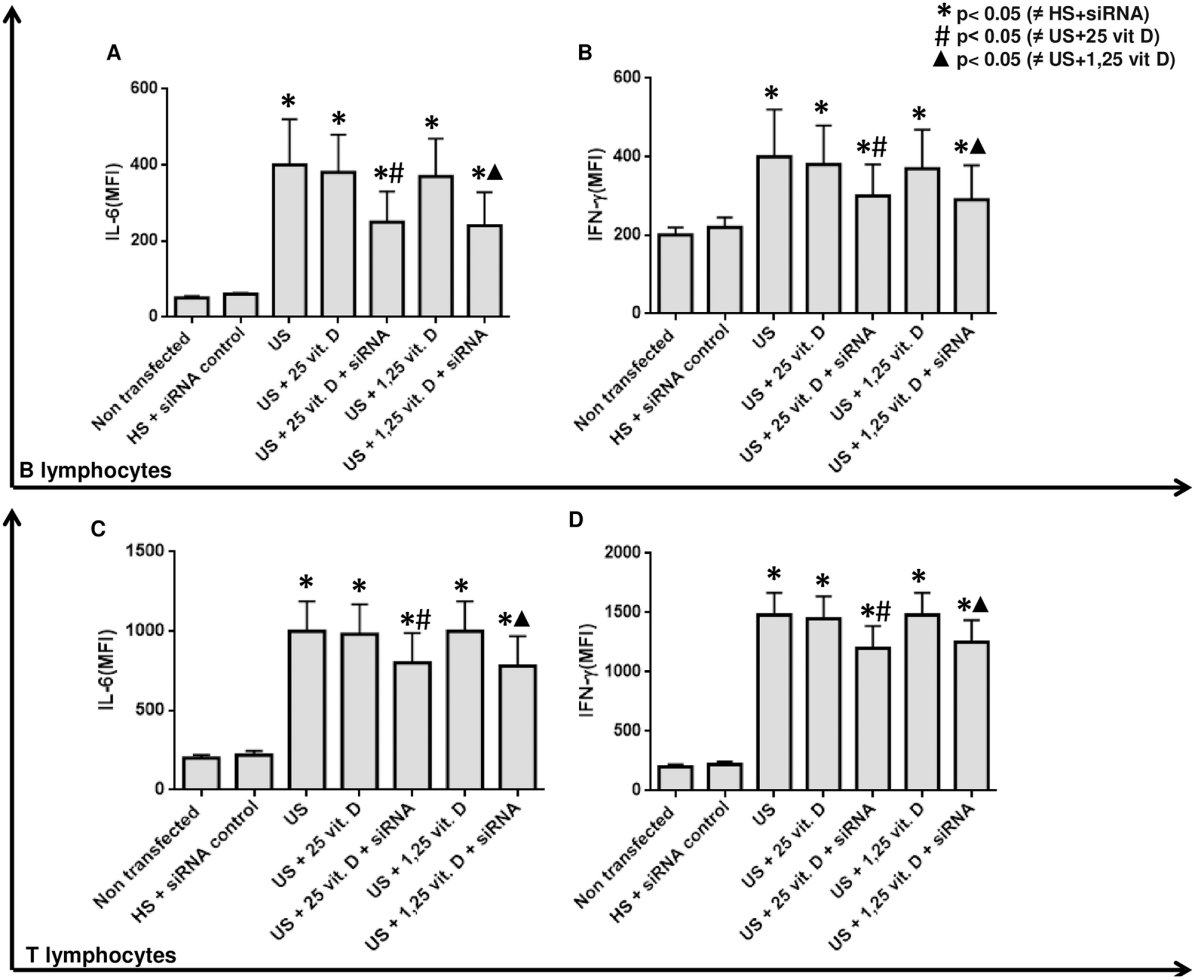
Effect of 25 and 1,25 vitamin D on IL-6 (MIF) and IFN-γ expression (MFI) in B lymphocytes (Figs A and B) and T lymphocytes (Figs C and D) in presence of healthy or uremic serum (US) and after CYP24 silencing (siRNA).

**Fig 4:**
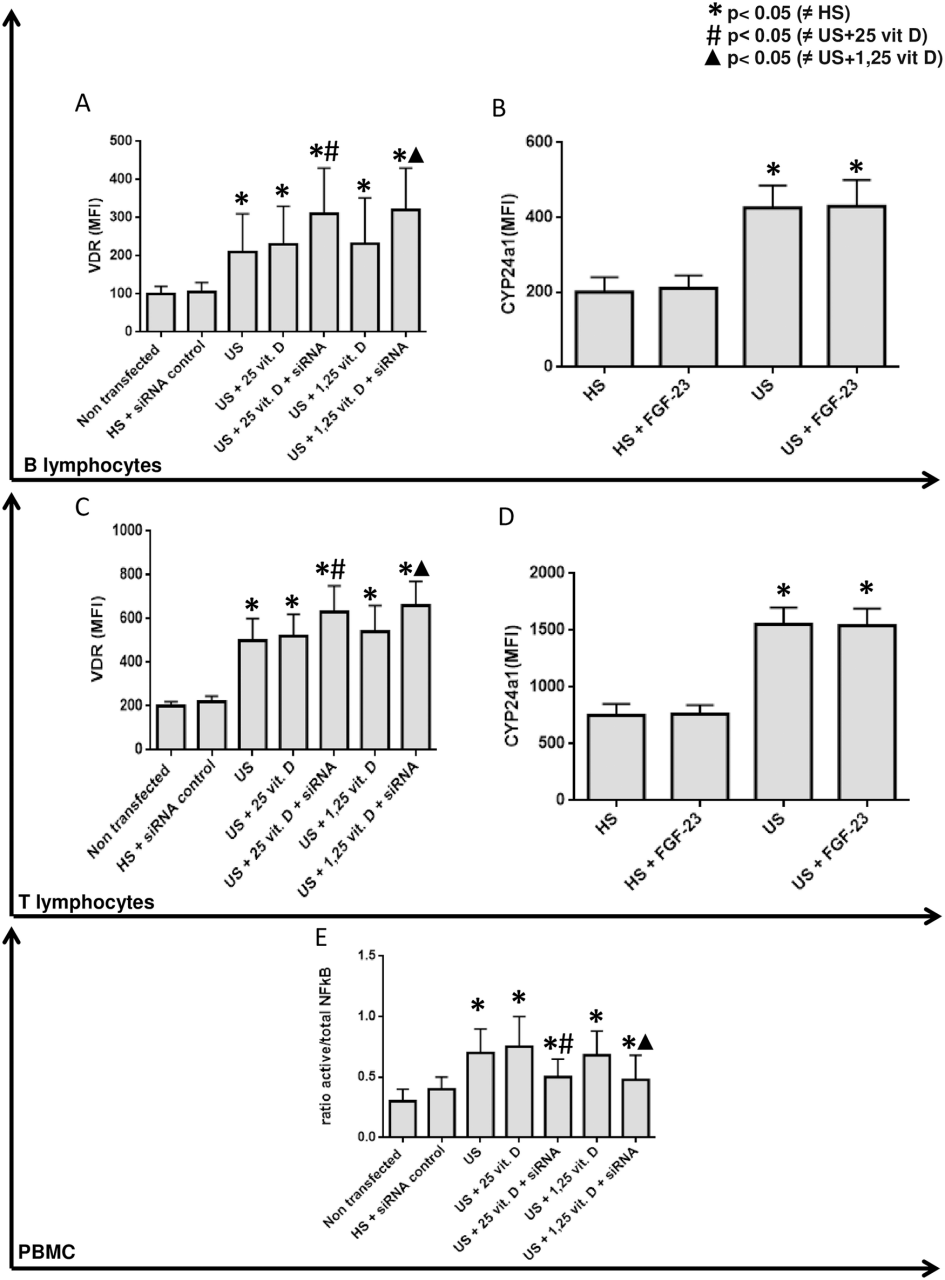
Effect of 25 and 1,25 vitamin D on VDR expression in B and T lymphocytes (Figs A and C) in the presence of healthy or uremic serum (US) and after CYP24 silencing (siRNA). CYP24 expression on healthy serum (HS); healthy serum+FGF-23 (HS+FGF-23) and uremic serum+FGF-23 incubation (US+FGF-23) (MFI) in B lymphocytes (Fig **B**) and T lymphocytes (Fig **D**). **E**: Effect of 25 and 1,25 vitamin D on NF-κB activation in PBMC in the presence of healthy or uremic serum **(US)** and after CYP24 silencing (siRNA).

As uremic serum increased CYP24a1 and this condition might influence the intracellular degradation of 1,25 vitamin D, we performed a CYP24a1 silencing. After this silencing plus treatment with both analogs of vitamin D we observed an additional effect of 25 and 1,25 vitamin D in the inhibition of TLR7, TLR9 (Fig 2), IL-6 and IFN-γ (Fig 3) and in the stimulation of VDR (Fig 4) expression on B and T lymphocytes compared to the effect of vitamin D without silencing CYP24a1.

As FGF-23 could modulate CYP24a1, we measured FGF-23 but did not observe an effect of FGF-23 on the expression of CYP24a1 in B and T lymphocytes in the presence or absence of uremic serum (Fig 4).

We observed that uremic serum induced a high ratio of NF-κB activation which is reduced with vitamin D treatment and CYP24a1 silencing (Fig 4).

## Discussion

This is the first randomized, placebo-controlled study that showed that cholecalciferol supplementation has an anti-inflammatory effect and improves vitamin D intracellular regulatory enzymes in lymphocytes in the uremic environment. We thereby focused on lymphocytes, as these cells are critical components of the adaptive immune system and to the best of our knowledge, we are the first to report the impact of vitamin D on B and T lymphocytes in uremia. To confirm our pilot clinical results and to better understand the mechanism of vitamin D, we performed an *in vitro* model using lymphocytes from healthy volunteers incubated with a uremic pool serum.

In the present clinical trial, we showed that cholecalciferol treatment was efficient to normalize the serum levels of 25(OH)D and decrease PTH levels in patients supplemented compared to placebo group, suggesting that adequacy of vitamin D status might have modulated synthesis this hormone in parathyroid cells and this effect might have contributed to lower bone reabsorption [12]. In respect to the serum FGF-23 in cholecalciferol group, the significant difference detected by the group-time interaction analysis might be due to the higher production of this hormone by osteocytes, since vitamin D administration upregulates FGF-23 [13, 28].

In addition to the classical action of vitamin D, the 25 vitamin D repletion by cholecalciferol supplementation caused a reduction in the intracellular expression of TLR7, TLR9 and IFN-γ in B and T lymphocytes from dialysis patients. These data demonstrate that 25 vitamin D repletion has an immunomodulatory effect on lymphocytes independent of renal 1,25 vitamin D synthesis, as was already reported in our recently study with monocytes. In agreement with these results, we observe similar results after *in vitro* incubation with 1,25 and 25 vitamin D on lymphocytes TLR and cytokines expression. As 25 vitamin D requires intracellular conversion into an active form to present biological activity [6, 29], these results support that lymphocytes have the ability to convert 25 to 1,25 vitamin D.

The mechanism by which vitamin D reduces inflammation remains poorly understood. The supplementation with cholecalciferol also increased the expression of VDR in lymphocytes from dialysis patients. This result has been reported in others cell types [25,26], but our study is the first to report it in lymphocytes from CKD patients. Previous studies supported that anti-inflammatory action of vitamin D is regulated in part by VDR activation which blocks NF-κB activation [30–32]. It has been shown that vitamin D down-regulates NF-κB activation by increasing the expression of iκB in the cells, thus interfering with the nuclear translocation of the activated NF-κB subunits. *In vitro*, we found that NF-κB was activated by uremic serum pool and it was reduced after vitamin D treatment [30–32]. Taken together, these results suggest that the anti-inflammatory effects of vitamin D occur in part by modulation of the NF-κB pathway and it may cause a decline in intracellular synthesis of inflammatory cytokines, including IFN-γ and IL-6 in lymphocytes, as described previously in monocytes [29, 33].

In the present study, we did not fully investigate the mechanism of vitamin D, but we hypothesized that a higher availability of 25 vitamin D for extra-renal tissues might result in increased activation of VDR, enhanced expression of CYP27b1 and increased intracellular conversion of 25 to 1,25 vitamin D, which in turn may down-regulate important mediators in inflammatory signaling, such as TLRs and cytokines by NF-κB reduced activation [30–32]. In fact, we observed that there was a negative correlation between VDR and TLR7, TLR9 and IFN-γ expression in lymphocytes from dialysis patients and a reduction in NF-κB activation together with a rise in VDR expression after the *in vitro* incubation of 25 and 1,25 vitamin D in lymphocytes from healthy volunteers exposed to uremic serum pool.

The next significant finding was the observed local decline in lymphocytes CYP24a1 and an up-regulation in CYP27b1 expression after cholecalciferol treatment, which is not consistent with the known effect of 1,25 vitamin D in renal tubular cells [9, 16]. In this regard, PTH increases the activity of CYP27b1, resulting in increased production of 1,25 vitamin D, and 1,25 vitamin D stimulates FGF-23 secretion, which in turn attenuates renal production of 1,25 vitamin D by inhibiting expression of CYP27b1 and by simultaneously increasing expression of CYP24a1 [9, 34, 35]. Finally, local production of 1,25 vitamin D cause up-regulation of renal CYP24 activity [16, 36]. Therefore, as consequence of 25 vitamin D repletion, we expected to be elicited a decline in CYP27b1 and an up-regulation of CYP24a1 expression. Since we did not observe a correlation of these enzymes from CKD patients and PTH or FGF-23, the exact regulation of the local metabolism of vitamin D in lymphocytes from CKD remains uncertain. More recently, activation of the TLR2 and TLR4 has been shown to up-regulate the expression of CYP27b1 and VDR genes in monocytes and macrophages, indicating the important role played by cytokines in producing the active form of vitamin D in these cells [37, 38]. Further studies are needed to evaluate the regulation of CYP27b1 and CYP24a1 by TLR activation on leukocytes in the uremic environment.

The last important observation is that the *in vitro* incubation with 25 vitamin D demonstrated that concentrations at 30 ng/ml did not achieve an efficient anti-inflammatory response of human lymphocytes. However, in the present study, an interesting finding was the higher expression of CYP24a1 in the presence of uremic serum. This finding could explain the reason for which 25 and 1,25 vitamin D treatment did not decrease the inflammatory mediators in an efficient manner on lymphocytes. The elevated expression of CYP24a1 could result in an elevated catabolism of intracellular 1,25 vitamin D and result in a resistance to response [14, 39] of lymphocyte by anti-inflammatory effects of vitamin D. To support this hypothesis, intracellular concentrations of 1,25 vitamin D should have been measured. However, until the moment, there is not a gold standard technical to measure intracellular 1,25 vitamin D. To evaluate this mechanism, we silenced CYP24a1 expression and incubated lymphocytes with 25 and 1,25 vitamin D.

The CYP24a1 silencing resulted in a decrease in IL-6, IFN-γ, TLR7 and TLR9 expression on B and T lymphocytes and an increase in VDR expression compared to the treatment with 1,25 or 25 vitamin D alone. These results indirectly support that CYP24a1 might also play an important role in the regulation of the intracellular conversion of 1,25 vitamin D and this may have an impact on inflammatory mechanisms, at least in lymphocyte responses in a uremic environment. In this way, the main effect of both 25 and 1,25 vitamin D in decreased expression of inflammatory markers observed with siRNA-mediated silencing of CYP24a1 gene expression in lymphocytes, suggest that CYP24a1 should be investigated as pathway enrolled in negative immunomodulation exert by vitamin D. Unfortunately, we could not measure intracellular levels of 1,25 vitamin D to confirm this hypothesis.

To understand the cause of elevated expression of CYP24a1 and based on literature data [11, 28], we hypothesized that FGF-23 might be involved in this deregulation since is unclear whether FGF-23 could regulate vitamin D enzymes in lymphocytes. However, we did not find an association between FGF-23 and these vitamin D regulatory enzymes in circulating lymphocytes from dialysis patients. Additionally, *in vitro*, we did not observe any effect of FGF-23 on vitamin D regulatory enzymes in lymphocytes, differently as described in kidney cells [11, 28]. Thus, these results suggest that FGF-23 do not have an impact in these vitamin D regulatory enzymes in lymphocytes.

In conclusion, this is the first randomized, placebo-controlled study that showed that cholecalciferol supplementation has an anti-inflammatory effect and improves vitamin D intracellular regulatory enzymes in lymphocytes in the uremic environment. However, the current study has no data to support whether the effect of vitamin D on reduction of inflammation in lymphocytes may have an impact on systemic levels of inflammatory mechanisms or on clinical outcomes. Further studies are needed to evaluate this condition in uremic patients.

## Supporting information

S1 Fig Flow cytometric analysis of B and T subsets. Whole blood was stained with anti-CD19 PerCP, anti-CD3 FITC. CD19+ cells and CD3+ cells were gated for further analysis of B cells for TLR7, TLR9, IL-6, IFN-γ, VDR, CYP27b1 and CYP24a1 by Mean of Fluorescence Intensity (MFI) from histograms plots.
(PDF)

S1 Table Expression of TLR7, TLR9, IFN-γ, IL-6, VDR, CYP27b1 e CYP24a1 in B and T lymphocytes between peritoneal dialysis (PD) and hemodialysis (HD) patients. (PDF)

S2 Table Demographic data. (PDF)

S3 Table Correlation between TLR7, TLR9, IFN-γ, FGF-23, CYP27b1 and CYP24a1. (PDF)

S4 Table Expression of TLR7, TLR9, IL-6, IFN-γ, VDR, CYP27b1 and CYP24a1 in B and T lymphocytes in the presence of 25 or 1,25 vitamin D incubated with uremic serum compared with healthy serum. (PDF)

S1 Raw Data Raw Data Excel spreadsheet of raw data from the study. (XLSX)

S1 CONSORT Checklist Completed CONSORT checklist for randomized controlled trials referencing the manuscript. (DOC)

S1 Protocol Design and implementation of the project. (DOC)
