# Supplementary figures and images for: Cholecalciferol decreases inflammation and improves vitamin D regulatory enzymes in lymphocytes in the uremic environment: A randomized controlled pilot trial
Source: PLoS One. 2017 Jun 30;12(6):e0179540. doi: 10.1371/journal.pone.0179540 (PMC5493305; doi:10.1371/journal.pone.0179540)

1A

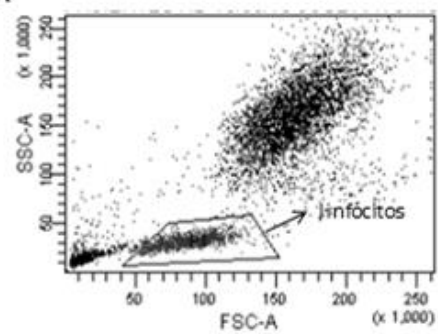

1B

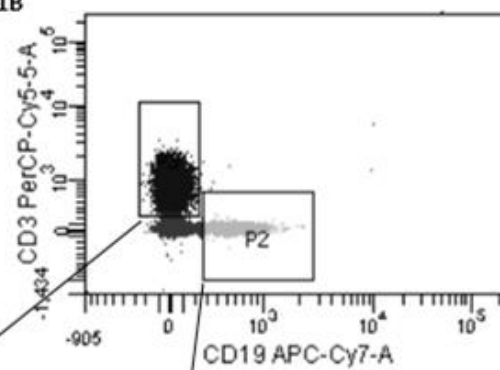

1C

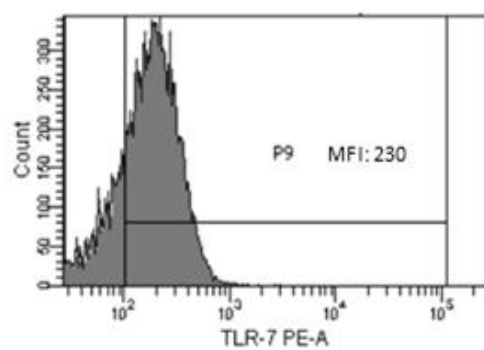

1D

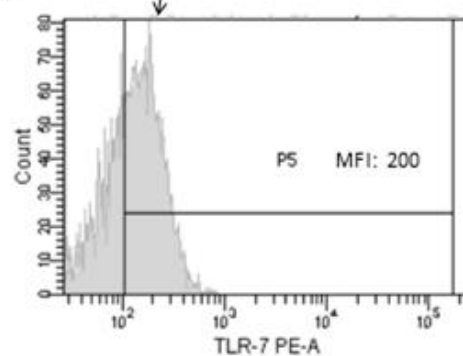

Supplement: S1 Fig — Whole blood was stained with anti-CD19 PerCP, anti-CD3 FITC. CD19+ cells and CD3+ cells were gated for further analysis of B cells for TLR7, TLR9, IL-6, IFN-γ, VDR, CYP27b1 and CYP24a1 by Mean of Fluorescence Intensity (MFI) from histograms plots. (PDF) [file pone.0179540.s001.pdf]
