# Supplementary material for: Cholecalciferol decreases inflammation and improves vitamin D regulatory enzymes in lymphocytes in the uremic environment: A randomized controlled pilot trial
Source: PLoS One. 2017 Jun 30;12(6):e0179540. doi: 10.1371/journal.pone.0179540 (PMC5493305; doi:10.1371/journal.pone.0179540)
Supplement: S1 Table — (PDF) [file pone.0179540.s002.pdf]

S1 Table. Expression of TLR7, TLR9, IFN-γ, IL-6, VDR, CYP27b1 e CYP24a1 in B and T lymphocytes between peritoneal dialysis (PD) and hemodialysis (HD) patients

	PD (n=16)	HD (n=16)	p
B lymphocytes			
TLR7	262.6±62.5	315.2±52.5	0.23
TLR9	1483.4±480	1531.4±520	0.44
IFN-γ	685.5±350	754.4±320	0.63
IL-6	291.2±120	219.8±140	0.54
VDR	812.5±340	801.5±360	0.43
CYP27b1	311.2±108	253.4±123	0.26
CYP24a1	269.5±111	256.6±98	0.36
T lymphocytes			
TLR7	414.6±79.2	320.2±79.2	0.33
TLR9	1432.1±595	1508.6±580	0.46
IFN-γ	768.5±395.5	881.2±405	0.33
IL-6	265.5±110.8	243.4±115.6	0.44
VDR	708.7±237.7	722.4±230	0.61
CYP27b1	310.6±90	269.7±100	0.62
CYP24a1	283.4±100	213.3±88	0.33

Mean ± SD or Median and Interquartil or n (%)
Mann-Whitney
