# Supplementary material for: Cholecalciferol decreases inflammation and improves vitamin D regulatory enzymes in lymphocytes in the uremic environment: A randomized controlled pilot trial
Source: PLoS One. 2017 Jun 30;12(6):e0179540. doi: 10.1371/journal.pone.0179540 (PMC5493305; doi:10.1371/journal.pone.0179540)
Supplement: S2 Table — (PDF) [file pone.0179540.s003.pdf]

S2 Table. Demographic data

	Placebo (n=22)	Cholecalciferol (n=21)	P
Age (years)	58±14	53±13	0.4
Gender (% men)	52.4	54.5	0.7
Ethnic (% white)	60.6	62	0.7
Etiology of CKD (%)			0.7
Diabetes Mellitus	14	20.9	
Hypertension	2.3	2.3	
Polycystic kidneys	11.6	5	
Unknown	11.6	16.3	
Others	9.3	7	
Duration of dialysis (months)	43 (20-75)	51 (18-78)	0.6
Season (%)			0.8
Summer/Fall	42.9	37.3	
Winter/Spring	57.1	62.7	

Mean ± SD or Median and Interquartil or n (%)

Mann-Whitney or chi-square test (categorical variables).
