# Supplementary material for: Cholecalciferol decreases inflammation and improves vitamin D regulatory enzymes in lymphocytes in the uremic environment: A randomized controlled pilot trial
Source: PLoS One. 2017 Jun 30;12(6):e0179540. doi: 10.1371/journal.pone.0179540 (PMC5493305; doi:10.1371/journal.pone.0179540)
Supplement: S3 Table — (PDF) [file pone.0179540.s004.pdf]

S3 table. correlation between TLR7, TLR9, IFN-γ, PTH, FGF-23, VDR, CYP27b1 and CYP24a1

	VDR	CYP27b1	CYP24a1
**TLR7**	**r= -0.48 p= 0.02**	r= 0.29 p= 0.19	r= 0.10 p= 0.90
**TLR9**	**r= -0.45 p= 0.04**	r= 0.40 p= 0.46	r= 0.37 p= 0.58
**IFN-γ**	**r=-0.72 p= 0.03**	r= 0.02 p= 0.39	r= 0.43 p= 0.51
**PTH**		r= 0.28 p= 0.40	r= 0.37 p= 0.31
**FGF-23**		r= 0.33 p= 0.50	r= 0.12 p= 0.41

Pearson correlation
