# Supplementary material for: Cholecalciferol decreases inflammation and improves vitamin D regulatory enzymes in lymphocytes in the uremic environment: A randomized controlled pilot trial
Source: PLoS One. 2017 Jun 30;12(6):e0179540. doi: 10.1371/journal.pone.0179540 (PMC5493305; doi:10.1371/journal.pone.0179540)
Supplement: S4 Table — (PDF) [file pone.0179540.s005.pdf]

S4 Table. Expression of TLR7 , TLR9 , IL-6 , IFN-γ , VDR , CYP27b1 and CYP24a1 in B and T lymphocytes in the presence of 25 or 1,25 vitamin D incubated with uremic serum compared with healthy serum

	HEALTHY SERUM (HS)				UREMIC SERUM (US)				
	HS	$25(OH)D_3$	$1,25(OH)_2D_3$	p●	US	$25(OH)D_3$	$1,25(OH)_2D_3$	p#	p*
B lymphocytes									
TLR7	54 (41 – 67)	39 (37 - 41)	37 (24 – 50)	0.52	421* (320 – 500)	412 (295 – 540)	406 (329 – 530)	0.46	**0.03**
TLR9	81 (60 – 104)	45 (15 – 80)	58 (20 – 92)	0.28	936* (420 – 1400)	876# (360 – 1380)	870# (420 – 1290)	**0.03**	**0.007**
IFN-γ	61 (48 - 74)	40 (18 – 68)	45 (27 – 63)	0.44	350* (300 – 400)	325 (245 – 405)	315 (246 – 384)	0.57	**0.001**
IL-6	101 (97 – 105)	74 (50 - 98)	65 (15 – 85)	0.88	892* (560 – 1200)	778# (400 – 1200)	820# (580 – 1100)	**0.04**	**0.02**
VDR	470 (234 - 680)	515 (240 – 1240)	500 (300 – 1020)	0.34	1500* (800 – 1900)	1567 (850 - 2200)	1585 (880 - 2300)	0.66	**0.001**
CYP27b1	1800 (510 – 3130)	2015 (815 - 3215)	1910 (710 – 3110)	0.23	5202* (3400 - 6030)	5550 (3700 – 7300)	5257 (3900 - 7700)	0.33	**0.007**
CYP24a1	1050 (550 - 1650)	990 (490 - 1480)	950 (450 - 1450)	0.51	3343* (1940 - 4740)	2953 (1550 - 4350)	3045 (1650 – 4450)	0.67	**0.001**
T lymphocytes									
TLR7	40 (27 – 53)	25 (22 – 28)	25 (10 – 40)	0.34	353* (120 – 580)	318# (120 – 520)	290# (77 – 503)	**0.02**	**0.03**
TLR9	65,(42 – 88)	60 (22 – 98)	60 (22 – 98)	0.23	533* (194-863)	444# (194 – 640)	192# (117 – 267)	**0.03**	**0.007**
IFN-γ	44(31 – 57)	42 (20 – 64)	42 (20 – 64)	0.51	245* (145 – 345)	224 (117 – 330)	219 (119 – 319)	0.24	**0.001**
IL-6	30 (26 – 34)	28 (18 – 48)	28 (16 – 46)	0.88	492* (192 – 792)	379 (100 – 660)	354(50 - 650)	0.26	**0.02**
VDR	300(143 -387)	320 (200 - 470)	330 (190 - 470)	0.34	434* (314 – 554)	438 (300 - 580)	438 (292 - 588)	0.41	**0.001**
CYP27b1	790(640 - 1077)	883 (633 – 1136)	825 (605 – 1045)	0.23	1076* (750 - 1320)	1010 (680 - 1450)	1005 (575 - 1200)	0.45	**0.007**
CYP24a1	880(484 - 1363)	800 (420 - 1184)	780 (460 - 1080)	0.51	1740* (1325 - 2235)	1669 (990 - 2050)	1643 (1110 - 2233)	0.32	**0.001**

p● 25(OH)D and 1,25(OH)D versus HS (control)
p# 25(OH)D and 1,25(OH)D versus US
p* US versus HS
