# Supplementary material for: Cholecalciferol decreases inflammation and improves vitamin D regulatory enzymes in lymphocytes in the uremic environment: A randomized controlled pilot trial
Source: PLoS One. 2017 Jun 30;12(6):e0179540. doi: 10.1371/journal.pone.0179540 (PMC5493305; doi:10.1371/journal.pone.0179540)

**UNIVERSIDADE FEDERAL DE SÃO PAULO - UNIFESP**

**DEPARTAMENTO DE MEDICINA - DISCIPLINA DE NEFROLOGIA**

**Effect of Cholecalciferol on inflammation and vitamin D regulatory enzymes in lymphocytes in uremic environment**

**SÃO PAULO**

**2012**

**Abstract**

Cardiovascular (CV) and infectious diseases remain the most common causes of mortality and morbidity, respectively among patients with chronic kidney disease (CKD). Previous studies have reported several alterations in immune cells, which contribute to increase incidence of CV and infectious diseases observed in this population. Biocompatibility of dialysis membrane and uremic toxins are the main factors associated with immune dysfunction in uremia. Furthermore, the Vitamin D deficiency is also associated with immunologic co-morbidities that are present in CKD. Vitamin D deficiency is highly prevalent among patients with all stages of CKD, but it is greater in peritoneal dialysis (PD) patients than the hemodialysis (HD) and pre-dialysis [CKD](http://www.ncbi.nlm.nih.gov/pubmed/22074789) patients. As the vitamin D receptor is expressed in the immune cells (B cells, T cells and others), and these cells are able to synthesizing the active vitamin D metabolite, many studies have investigated the role of vitamin D in the immune system. In this regard, previous studies have reported that vitamin D inhibits the TLR-induced cytokines production, including IFN-γ and IL-6. Thus, the purpose or this study is evaluate the effect of cholecalciferol treatment therapy on apoptosis, IFN-γ and IL-6 production, TLR-7 and TLR-9 expression in lymphocytes B *in vitro* and *in vivo.*

**Key Words:** Cholecalciferol, lymphocytes B, chronic kidney disease

INTRODUCTION

Background and Scientific Rationale

Chronic Kidney Disease (CKD) patients have a higher prevalence in the rate of cardiovascular diseases (CVD) and infections, representing the main causes of mortality and morbidity, respectively, in this population.^(1-2)^ In the last years epidemiological studies have observed an association between hypovitaminosis D with a higher prevalence in infections, CVD and mortality in patients with CKD.^(3,4,5,6)^

The active form of vitamin D (1,25 dihydroxyvitamin D or calcitriol) is synthesized from cholecalciferol, an inactive form. (13) There are two sources of cholecalciferol in the body: it is ingested through feeding or synthesized in the skin from the 7-dehydrocholesterol (pro-vitamin D3), a photolysis reaction that occurs after exposure to sunlight due to ultraviolet (UVB) photons. After photo-synthesis, pre-vitamin D3 can be thermally isomerized to cholecalciferol (vitamin D3), which, via vitamin D binding protein (DBP), enters the circulation. (14) In the liver, vitamin D3 undergoes the first Hydroxylation by the action of the 25-hydroxylase enzyme present in the mitochondria of hepatocytes, forming 25-hydroxyvitamin D [25 (OH) D3] or calcidiol, which is the most abundant form in the body. In order for vitamin D to become biologically active, however, a second hydroxylation that occurs in the kidney is required by the action of the 1-α-hydroxylase enzyme present in the mitochondria of the proximal renal tubule cells, ultimately resulting in the formation of 1,25-dihydroxyvitamin D [1,25 (OH) 2 D 3]. (15) É importante notar que a dosagem da forma 25(OH)D3 (calcidiol) representa a ferramenta mais fidedigna para a aferição do nível de vitamina D no organismo, pois é a principal forma circulante, sendo sua concentração plasmática 1000 vezes maior do que a forma ativa (1,25(OH)_2_D3), e por apresentar meia vida na circulação de aproximadamente duas semanas, enquanto a forma ativa da vitamina D possui meia vida de apenas 4 horas. ^(16,17)^

Regarding the supplementation with Colecalciferol, administration of the 25 (OH) D3 form has also been more widely used because it has a higher plasma half-life and therefore a higher bioavailability. (18,19) In addition, treatment with the 25 (OH) D3 form shows less hypercalcemia when compared to treatment with the 1,25 (OH) 2 D3 form. (18,19) Thus, treatment with 25 (OH) D3 form has advantages compared to the active form, since it has lower adverse effects and greater bioavailability. Although patients with CKD have a 25 (OH) D 3-form conversion to 1,25 (OH) 2 D 3 form, recent studies have shown that treatment with 25 (OH) D3 was sufficient to raise the serum concentration of the active form (1 , 25 (OH) 2 D3), but it is not yet clear whether this increase occurs through extra-renal synthesis or renal residual function. (18, 19, 20)

It has recently been found that cells of the immune system express vitamin D receptors (21) and present enzymatic machinery to convert to 25 (OH) D 3 in its active form 1,25 (OH) 2 D 3 (22,23) and, with this , He began to explore his immunomodulatory role. VDR is a member of the superfamily of nuclear hormone receptors. The interaction with 1,25- (OH) 2-cholecalciferol leads to conformational changes in these receptors, increasing affinity with another cytosolic receptor called the retinoic receptor X (RXR), forming a heterodimer (VDR-RXR). This heterodimer translocates to the nucleus, and alters gene expression in many cell types (25,26), including cells of the immune system, such as B lymphocytes, in which case they can modulate cytokines. (22,24)

A decrease in VDR expression has been demonstrated in diseases characterized by a pro-inflammatory state, such as diabetes, CVD and CKD (18, 27, 28). Stubbs et al. Observed that cholecalciferol therapy resulted in increased VDR expression in monocytes from hemodialysis patients, and reduced in levels of (IL-6), interleukin-8 (IL-8), and Tumor Necrosis Factor α (TNF-α), respectively, have been shown to increase the expression of VDR in monocytes from patients on hemodialysis and to reduce circulating levels of interleukin-6 (TNF-α) (18). In addition, Khoo et al 2011 also observed that vitamin D inhibits the synthesis of pro-inflammatory cytokines in vitro. An important finding in this study was that this effect was attenuated in the presence of the VDR antagonist. Validating the hypothesis that the immunomodulatory effect of vitamin D is mediated by signaling pathways recruited by VDR (29).

In respt to 1α-hydroxylase, Viaene et al (2012) recently observed that incubation of FGF-23 (currently classified as uremic toxin) decreased the expression of this enzyme in monocytes, which may lead to less synthesis of the active form of vitamin D locally 30). Most studies conducted in the literature evaluating the immunomodulatory effect of vitamin D are performed on monocytes, but B lymphocytes also express VDRs and enzymes involved in vitamin D metabolism (24). In fact, an important role of 25(OH)D and 1,25(OH)2D3 in the immune system involves the control of inflammatory responses. An inverse correlation between serum 25(OH)D with C-reactive protein (CRP) and interleukin – 6 (IL-6) has been reported in CKD patients [6, 15, 16]. Recently, some studies investigated the impact of supplementation with cholecalciferol on systemic inflammation in CKD patients. Stubbs et al (2010) observed a reduction on serum TNF-α, IL-6 and CRP in hemodialysis patients after treatment with cholecalciferol [17]. Alvarez et al (2014 ) also observed a decrease in MCP -1 monocytes expression in patients in pre -dialysis stage (stages 2 and 3) that received cholecalciferol supplementation [18].

Toll-like receptors (TLR) represent an important pathway in the signaling of inflammatory responses. They recognize highly conserved structural motifs known as pathogen-associated microbial patterns (PAMPs), which are exclusively expressed by microbial pathogens, or danger-associated molecular patterns (DAMPs), which are endogenous molecules or non-pathogens products. In turn, activation of TLR by ligands leads to production of pro-inflammatory cytokines, via signaling pathways activating transcription factors, mainly NF-kappa-B-activating kinase (NF-κB) [19, 20].

The impact of renal dysfunction on TLR expression is poorly understood. Some studies have shown that patients on dialysis have low TLR4 expression on monocytes, resulting in a decreased ability to recognize and synthesize cytokines in response to a bacterial challenge [21]. In contrast, monocytes from pre-dialysis patients exhibit high expression of TLRs (TLR2 and TLR4) as well as high production of pro-inflammatory cytokines (TNF-α and IL-6) [22-24]. However, not only monocytes express TLRs. Leukocytes as B and T lymphocytes also express TLRs, but also it seems that these cells express only TLR7 and TLR9. Both are intracellular pattern-recognition receptors that recognize specific pathogen ligands, especially viral double-stranded RNA (as influenza and hepatitis virus) [19, 20, 24]. Once these viruses activate these receptors, a limited production of inflammatory cytokines occurs, as a physiologic response against these pathogens. In contrast, the upregulation of these receptors may result in increased production of these cytokines and more inflammation. However, there are few studies investigating the mechanisms of inflammatory responses on lymphocytes from CKD patients without viral infection [1, 25, 26]. Moreover, there are no reports that had investigated TLRs expression, VDR and vitamin D regulatory enzymes in these cells. It is also unclear whether vitamin D could modulate these receptors in a uremic environment

**Purpose**

Therefore, the purpose of this study will be to evaluate the effect of uremia before and after cholecalciferol supplementation on the expression of TLR7, TLR9, IFN-γ, IL-6, VDR, CYP27B1 and CYP24 in B and T lymphocytes from dialysis patients with hypovitaminosis D (in vivo study). We also will be assess the effect of 25(OH)D_3_ and 1,25 (OH)_2_D_3_ on lymphocytes from healthy volunteers (HV) in the presence or absence of uremic serum in respect of inflammation response mechanisms, intracellular VDR and vitamin D regulatory enzymes (in vitro study).

**In Vivo**

*Subjects and Methods*

Patients undergoing hemodialysis (HD) and peritoneal dialysis (PD) with age between 18-80 years, dialysis vintage of at least 3 months and 25(OH)D < 20 ng/mL were invited to participate in the study. The exclusion criteria were: use of any vitamin D compounds, glucocorticoids, immunosuppressors, history of liver failure, intestinal malabsorption, malignance, autoimmune disease, active infection, positive HIV, peritonitis in the last month or elevated serum ionized calcium (>1.40 mmol/L).

We expect that 180 subjects will be screened in order to randomize approximately 60 subjects

*Protocol*

At baseline, before starting a hemodialysis session, venous blood will be drawn for serum calcium, phosphorus, 25(OH)D and parathyroid hormone (PTH). Blood was drawn for 25(OH)D detection. Additionally, blood will draw for repeat calcium, phosphorus and PTH after 3 mounths. All blood will drawn before the next dose of cholecalciferol will given and directly before the start of a hemodialysis session.

Patients in the cholecalciferol group will receives 50,000 IU of cholecalciferol (1,000 IU/drop; Magister Pharmacy, São Paulo, SP, Brazil) twice a week, while the control group will be a placebo solution and were instructed to take 50 drops twice a week. Pharmaceutical presentation of the placebo and cholecalciferol were identical. To minimize the effects of vitamin D synthesis by the skin, all patients will use sunscreen (SPF 30) and will instructed to wear it during the study period. To evaluate the compliance to the protocol patients will request to bring the medication container in the follow up visits. It will expect that the volume finished in 6 weeks, when the patient received other container to complete the study. In addition, during the monthly visits and through phone calls patients were encouraged to follow the protocol.

*Methods*

Blood samples will collect after eight hours of fasting from all participants at baseline and after 12 weeks. Serum dosages of 25(OH)D (chemiluminescence immunoassay, Abbott, Germany), high-sensitivity C-reactive protein (immunoturbidimetric assay, Beckman Coulter Biomedical, Ireland), parathyroid hormone (chemiluminescence immunoassay, reference values​​: 10 to 69 pg/mL), alkaline phosphatase (calorimetric method, reference values​​: 35 to 104 U/L), phosphorus (colorimetric method, reference values​​: 2.3 to 4.3 mg/dL), ionized calcium (ion-selective electrode method, reference values: 1.11 to 1.40 mg/dL), albumin (bromocresol green), urea (enzymatic method) and creatinine (kinetic Jaffe colorimetric method) will be perform.

*25(OH)D detection*

Serum 25(OH)D levels will measure by radioimmunoassay using the Immuno-Diagnostics Kit. PTH 1–84 was measured by immunoradiometric assay using the DiaSorin N-tact PTH SP IRMA kit (DiaSorin Inc., MN). Serum calcium and phosphorus were measured by Roche Cobas Integra autoanalyzer (F Hoffmann-La Roche Ltd, Basel, Switzerland).

FGF*23 detection*

Serum concentrations of FGF23 will measure  using ELISA kits (R&D Systems) according to the manufacturer’s instructions. Respective inter- and intra-assay coefficients of variation were 4.1% and 5.9%.

**In Vitro**

*Methods*

Human peripheral blood mononuclear cells (PBMC) will isolated from heparinized, venous blood of healthy donors by Ficoll-Hypaque Sigma-Aldrich (St. Louis, MO) density gradient centrifugation. 1×10^6^ PBMCs were cultured in RPMI-1640 and antibiotics (100 U/ml penicillin and 100 g/ml streptomycin) in presence or absence of calcidiol (30ng/mL) or calcitriol (0.5ng/mL) Sigma-Aldrich (St. Louis, MO) with or without uremic serum (50%) for 24 h at 37˚C with 5% CO_2_. Follow-up the incubation monensin will add in cell culture to blocks the golgi

*Measurement of CYP27B1 of TLR-7, TLR-9, IFN-γ, IL-6, VDR, CYP27 and CYP24 by Flow Cytometric protocol (in vivo and in vitro)*

A total of 100 uL whole blood *(in vivo)* or 5×10^5^ PBMC *(in vitro)* collected in sodium heparin will incubate with 10uL of peridinin chlorophyll protein (PerCP)-labeled CD3 antibody and 5uL of PE-CY7-labeled CD19 antibody (BD Biosciences,San Diego, CA) for 15 min in the dark at room temperature. Cells will wash with PBS and centrifuge at 2000 rpm for 10 min. The supernatant will discard, and 1 ml of Cytofix/Cytoperm solution (BD Biosciences) will add to the tubes and incubate for 20 min at 4°C. After incubation, cells will wash twice with 1 ml of Perm/Wash buffer (BD Biosciences). After centrifugation, the supernatant will again discard and the pellet will resuspend in 50uL of PBS. Use of primary (Santa Cruz, CA) and secundary antibodies (Invitrogen) is necessary to VDR, CYP27b1, CYP24 staining. The primary antibodies is added at a concentration of 1μg/mL and secundary antibodies is added at a concentration of 3μg/mL. Specifications of secundary antibodies is Alexa-Fluor 488-conjugated anti-human Cyp24, Alexa-Fluor647-conjugated anti-human Cyp27 and APC-conjugated anti-human VDR.

Another aliquot of 100µL heparinized whole blood *(in vivo)* or 5×10^5^ PBMC *(in vitro)* is  incubate with antibodies specific for 2uL of fluorescein isothiocyanate (FITC)-labeled IL-6 antibody (BD Biosciences), 10 uL of phycoerythrin (PE)-labeled TLR-7 antibody (R&D System), 3 uL of APC-CY7-labeled IFN-y antibody (eBiosciences), 5 uL of APC-labeled TLR-9 antibody (BD Biosciences), after CD3 and CD19 staining, permeabilization and fixation as described above. After washing twice with Perm/Wash buffer, the supernatant is again discard and the final pellet iz resuspended in 300 uL of PBS with 1% sodium azide. The acquisition of samples will be performed by a flow cytometer (FacsCanto I, BD Biosciences, San Diego, CA, USA). For each sample, data from 30.000 cells will be collected and analyzed. Forward and side scatters were used to gate and exclude cellular debris and data are presented as mean fluorescence intensity peak (MFI) in CD3+ (T lymphocytes) and CD19+ (B lymphocytes) gated and expression of TLR-7, TLR9, IFN-y, IL-6, VDR, CYP27 and CYP24.

Statistical Analysis

Normality test are performed for all variables in the in vivo and in vitro study. Data will be expressed as mean ± standard deviation for variables with normal distribution, as median and interquartile range for skewed distribution, and as frequencies for categorical variables. **1º)** To the *in vivo* study analysis, Student´s t test or Pearson Chi-square test will be used to compare the baseline characteristics between the 2 groups, as appropriate. Comparisons between baseline and after 12 weeks will be performed using General linear model test or Wilcoxon signed-rank test and Mann-Whitney test, as appropriate. **2º)** To the *in vitro* study analysis, we used nonparametric ANOVA Kruskal-Wallis for comparisons between groups and testing Post-Hoc: Games-Howell to determine differences between groups. Pearson's test was used to analyze correlations between the parameters of the study. Statistical significance was defined as p<0.05. All statistical analyses will be conducted using Statistical Package for Social Sciences for Windows version 18.0 (SPSS Inc., Chicago, IL, USA).

1. ***REFERENCES***
2. [Bagdasarian N](http://www.ncbi.nlm.nih.gov/pubmed?term=Bagdasarian N%5BAuthor%5D&cauthor=true&cauthor_uid=22284380), [Heung M](http://www.ncbi.nlm.nih.gov/pubmed?term=Heung M%5BAuthor%5D&cauthor=true&cauthor_uid=22284380), [Malani PN](http://www.ncbi.nlm.nih.gov/pubmed?term=Malani PN%5BAuthor%5D&cauthor=true&cauthor_uid=22284380). Infectious complications of dialysis access device. [Infect Dis Clin North Am.](http://www.ncbi.nlm.nih.gov/pubmed/22284380) 2012 Mar;26(1):127-41.
3. [Chaykovska L](http://www.ncbi.nlm.nih.gov/pubmed?term=Chaykovska L%5BAuthor%5D&cauthor=true&cauthor_uid=21888009), [Tsuprykov O](http://www.ncbi.nlm.nih.gov/pubmed?term=Tsuprykov O%5BAuthor%5D&cauthor=true&cauthor_uid=21888009), [Hocher B](http://www.ncbi.nlm.nih.gov/pubmed?term=Hocher B%5BAuthor%5D&cauthor=true&cauthor_uid=21888009). Biomarkers for the prediction of mortality and morbidity in patients with renal replacement therapy. [Clin Lab.](http://www.ncbi.nlm.nih.gov/pubmed/21888009) 2011;57(7-8):455-67
4. Wolf M, Shah A, Gutierrez O, Ankers E, Monroy M, Tamez H, Steele D, Chang Y, Camargo CA Jr, Tonelli M, Thadhani R: Vitamin D levels and early mortality among incident hemodialysis patients. Kidney Int 2007; 72: 1004–1013.
5. [Pecovnik-Balon B](http://www.ncbi.nlm.nih.gov/pubmed?term=Pecovnik-Balon B%5BAuthor%5D&cauthor=true&cauthor_uid=19695057), [Jakopin E](http://www.ncbi.nlm.nih.gov/pubmed?term=Jakopin E%5BAuthor%5D&cauthor=true&cauthor_uid=19695057), [Bevc S](http://www.ncbi.nlm.nih.gov/pubmed?term=Bevc S%5BAuthor%5D&cauthor=true&cauthor_uid=19695057), [Knehtl M](http://www.ncbi.nlm.nih.gov/pubmed?term=Knehtl M%5BAuthor%5D&cauthor=true&cauthor_uid=19695057), [Gorenjak M](http://www.ncbi.nlm.nih.gov/pubmed?term=Gorenjak M%5BAuthor%5D&cauthor=true&cauthor_uid=19695057). Vitamin D as a novel nontraditional risk factor for mortality in hemodialysis patients. [Ther Apher Dial.](http://www.ncbi.nlm.nih.gov/pubmed/19695057) 2009 Aug;13(4):268-72
6. [Inaguma D](http://www.ncbi.nlm.nih.gov/pubmed?term=Inaguma D%5BAuthor%5D&cauthor=true&cauthor_uid=18180871), [Nagaya H](http://www.ncbi.nlm.nih.gov/pubmed?term=Nagaya H%5BAuthor%5D&cauthor=true&cauthor_uid=18180871), [Hara K](http://www.ncbi.nlm.nih.gov/pubmed?term=Hara K%5BAuthor%5D&cauthor=true&cauthor_uid=18180871), [Tatematsu M](http://www.ncbi.nlm.nih.gov/pubmed?term=Tatematsu M%5BAuthor%5D&cauthor=true&cauthor_uid=18180871), [Shinjo H](http://www.ncbi.nlm.nih.gov/pubmed?term=Shinjo H%5BAuthor%5D&cauthor=true&cauthor_uid=18180871), [Suzuki S](http://www.ncbi.nlm.nih.gov/pubmed?term=Suzuki S%5BAuthor%5D&cauthor=true&cauthor_uid=18180871), [Mishima T](http://www.ncbi.nlm.nih.gov/pubmed?term=Mishima T%5BAuthor%5D&cauthor=true&cauthor_uid=18180871), [Kurata K](http://www.ncbi.nlm.nih.gov/pubmed?term=Kurata K%5BAuthor%5D&cauthor=true&cauthor_uid=18180871). Relationship between serum 1,25-dihydroxyvitamin D and mortality in patients with pre-dialysis chronic kidney disease. [Clin Exp Nephrol.](http://www.ncbi.nlm.nih.gov/pubmed/18180871) 2008 Apr;12(2):126-31
7. [Hansen D](http://www.ncbi.nlm.nih.gov/pubmed?term=Hansen D%5BAuthor%5D&cauthor=true&cauthor_uid=20003863), [Brandi L](http://www.ncbi.nlm.nih.gov/pubmed?term=Brandi L%5BAuthor%5D&cauthor=true&cauthor_uid=20003863), [Rasmussen K](http://www.ncbi.nlm.nih.gov/pubmed?term=Rasmussen K%5BAuthor%5D&cauthor=true&cauthor_uid=20003863). Vitamin D, cardiovascular disease and mortality in patients with kidney disease [Ugeskr Laeger.](http://www.ncbi.nlm.nih.gov/pubmed/20003863) 2009 Dec 7;171(50):3684-9.
8. Cuppari L, Carvalho AB, Draibe SA: Vitamin D status of chronic kidney disease patients living in a sunny country. J Ren Nutr 2008; 18(5):408-14.
9. Mehrotra R, Kermah D, Budoff M, et al . Hypovitaminosis D in chronic kidney disease. Clin J Am Soc Nephrol 2008; 3(4):1144-51.
10. Del Valle E, Negri AL, Aguirre C, et al. Prevalence of 25(OH) vitamin D insufficiency and deficiency in chronic kidney disease stage 5 patients on hemodialysis. Hemodial Int 2007; 3:315-21.
11. Shah N, Bernadini J, Piraino B. Prevalence and correction of 25(OH) vitamin D deficiency in peritoneal dialysis patients. Perit Dial Int 2005; 25:362-6.
12. Elder GJ, Mackun K. 25-Hydroxyvitamin D deficiency and diabetes predict reduced BMD in patients with chronic kidney disease. J Bone Res 2006; 21:1778-84.
13. [Prytuła A](http://www.ncbi.nlm.nih.gov/pubmed?term=Prytuła A%5BAuthor%5D&cauthor=true&cauthor_uid=22081234), [Wells D](http://www.ncbi.nlm.nih.gov/pubmed?term=Wells D%5BAuthor%5D&cauthor=true&cauthor_uid=22081234), [McLean T](http://www.ncbi.nlm.nih.gov/pubmed?term=McLean T%5BAuthor%5D&cauthor=true&cauthor_uid=22081234), [Balona F](http://www.ncbi.nlm.nih.gov/pubmed?term=Balona F%5BAuthor%5D&cauthor=true&cauthor_uid=22081234), [Gullett A](http://www.ncbi.nlm.nih.gov/pubmed?term=Gullett A%5BAuthor%5D&cauthor=true&cauthor_uid=22081234), [Knott C](http://www.ncbi.nlm.nih.gov/pubmed?term=Knott C%5BAuthor%5D&cauthor=true&cauthor_uid=22081234), [Cantwell M](http://www.ncbi.nlm.nih.gov/pubmed?term=Cantwell M%5BAuthor%5D&cauthor=true&cauthor_uid=22081234), [Hassen K](http://www.ncbi.nlm.nih.gov/pubmed?term=Hassen K%5BAuthor%5D&cauthor=true&cauthor_uid=22081234), [Ledermann S](http://www.ncbi.nlm.nih.gov/pubmed?term=Ledermann S%5BAuthor%5D&cauthor=true&cauthor_uid=22081234), [Rees L](http://www.ncbi.nlm.nih.gov/pubmed?term=Rees L%5BAuthor%5D&cauthor=true&cauthor_uid=22081234), [Shroff R](http://www.ncbi.nlm.nih.gov/pubmed?term=Shroff R%5BAuthor%5D&cauthor=true&cauthor_uid=22081234). Urinary and dialysate losses of vitamin D-binding protein in children on chronic peritoneal dialysis. [Pediatr Nephrol.](http://www.ncbi.nlm.nih.gov/pubmed/22081234) 2012 Apr;27(4):643-9
14. Lehmann B, Rudolph T, Pietzsch J, Meurer M. Conversion of vitamin D3 to 1,25-dihydroxyvitamin D3 in human skin equivalents. Exper Dermatol 2000; 9:97- 103.
15. Prosser DE, Jones G. Enzymes involved in the activation and inactivation of vitamin D. Trends Biochem Sci 2004; 29:664-73.
16. Rochel, N.; Moras, D. Ligand binding domain of vitamin D receptors. *Curr. Top. Med. Chem.* 2006, *6*, 1229–1241.
17. [Holick MF](http://www.ncbi.nlm.nih.gov/pubmed?term=Holick MF%5BAuthor%5D&cauthor=true&cauthor_uid=15585788). Sunlight and vitamin D for bone health and prevention of autoimmune diseases, cancers, and cardiovascular disease. [Am J Clin Nutr.](http://www.ncbi.nlm.nih.gov/pubmed/15585788) 2004 Dec;80(6 Suppl):1678S-88S.
18. [Holick MF](http://www.ncbi.nlm.nih.gov/pubmed?term=Holick MF%5BAuthor%5D&cauthor=true&cauthor_uid=18400738), [Chen TC](http://www.ncbi.nlm.nih.gov/pubmed?term=Chen TC%5BAuthor%5D&cauthor=true&cauthor_uid=18400738). Vitamin D deficiency: a worldwide problem with health consequences.[Am J Clin Nutr.](http://www.ncbi.nlm.nih.gov/pubmed/18400738) 2008 Apr;87(4):1080S-6S.
19. [StubbsJR](http://www.ncbi.nlm.nih.gov/pubmed?term=Stubbs JR%5BAuthor%5D&cauthor=true&cauthor_uid=20007751), [IdicullaA](http://www.ncbi.nlm.nih.gov/pubmed?term=Idiculla A%5BAuthor%5D&cauthor=true&cauthor_uid=20007751), [SlusserJ](http://www.ncbi.nlm.nih.gov/pubmed?term=Slusser J%5BAuthor%5D&cauthor=true&cauthor_uid=20007751), [MenardR](http://www.ncbi.nlm.nih.gov/pubmed?term=Menard R%5BAuthor%5D&cauthor=true&cauthor_uid=20007751), [QuarlesLD](http://www.ncbi.nlm.nih.gov/pubmed?term=Quarles LD%5BAuthor%5D&cauthor=true&cauthor_uid=20007751).Cholecalciferol supplementation alters calcitriolresponsive monocyte proteins and decreasesinflammatory cytokines in ESRD.[J Am Soc Nephrol.](http://www.ncbi.nlm.nih.gov/pubmed?term=Cholecalciferol Supplementation Alters Calcitriol- Responsive Monocyte Proteins and Decreases Inflammatory Cytokines in ESRD) 2010 Feb;21(2):353-61.
20. Bucharles S, Barberato SH, Stinghen AEM, et al. Impact of Cholecalciferol treatment on biomarkers of inflammation and myocardial structure in hemodialysis patients without hyperparathyroidism. J Ren Nutr. 2012; 22(2)284-291.
21. Adams JS, Hewison M. Extrarenal expression of the 25-hydroxyvitamin D-1-hydroxylase. A. Bioche. Bioph. 523 (2012) 95–102
22. [Sterling KA](http://www.ncbi.nlm.nih.gov/pubmed?term=Sterling KA%5BAuthor%5D&cauthor=true&cauthor_uid=22614789), [Eftekhari P](http://www.ncbi.nlm.nih.gov/pubmed?term=Eftekhari P%5BAuthor%5D&cauthor=true&cauthor_uid=22614789), [Girndt M](http://www.ncbi.nlm.nih.gov/pubmed?term=Girndt M%5BAuthor%5D&cauthor=true&cauthor_uid=22614789), [Kimmel PL](http://www.ncbi.nlm.nih.gov/pubmed?term=Kimmel PL%5BAuthor%5D&cauthor=true&cauthor_uid=22614789), [Raj DS](http://www.ncbi.nlm.nih.gov/pubmed?term=Raj DS%5BAuthor%5D&cauthor=true&cauthor_uid=22614789). The immunoregulatory function of vitamin D: implications in chronic kidney disease. [Nat Rev Nephrol.](http://www.ncbi.nlm.nih.gov/pubmed/22614789) 2012 May 22;8(7):403-12.
23. Mora JR, Iwata M, von Andrian UH. Vitamin effects on the immune system: vitamins A and D take centre stage. Nat Rev Immunol 2008; 8:685-98.
24. [Human T lymphocytes are direct targets of 1,25-dihydroxyvitamin D3 in the immune system.](http://www.ncbi.nlm.nih.gov/pubmed/20302932) Baeke F, Korf H, Overbergh L, van Etten E, Verstuyf A, Gysemans C, Mathieu C. J Steroid Biochem Mol Biol. 2010 Jul;121(1-2):221-7.
25. [Heine G](http://www.ncbi.nlm.nih.gov/pubmed?term=Heine G%5BAuthor%5D&cauthor=true&cauthor_uid=18651709), [Niesner U](http://www.ncbi.nlm.nih.gov/pubmed?term=Niesner U%5BAuthor%5D&cauthor=true&cauthor_uid=18651709), [Chang HD](http://www.ncbi.nlm.nih.gov/pubmed?term=Chang HD%5BAuthor%5D&cauthor=true&cauthor_uid=18651709), [Steinmeyer A](http://www.ncbi.nlm.nih.gov/pubmed?term=Steinmeyer A%5BAuthor%5D&cauthor=true&cauthor_uid=18651709), [Zügel U](http://www.ncbi.nlm.nih.gov/pubmed?term=Zügel U%5BAuthor%5D&cauthor=true&cauthor_uid=18651709), [Zuberbier T](http://www.ncbi.nlm.nih.gov/pubmed?term=Zuberbier T%5BAuthor%5D&cauthor=true&cauthor_uid=18651709), [Radbruch A](http://www.ncbi.nlm.nih.gov/pubmed?term=Radbruch A%5BAuthor%5D&cauthor=true&cauthor_uid=18651709), [Worm M](http://www.ncbi.nlm.nih.gov/pubmed?term=Worm M%5BAuthor%5D&cauthor=true&cauthor_uid=18651709). 1,25-dihydroxyvitamin D(3) promotes IL-10 production in human B cells. [Eur J Immunol.](http://www.ncbi.nlm.nih.gov/pubmed?term=1%2C25-dihydroxyvitamin D3 promotes IL-10 production in human B cells) 2008 Aug;38(8):2210-8
26. Carlberg, C. 1996. The vitamin D3 receptor in the context of the nuclear re- ceptor superfamily : the central role of the retinoid X receptor. Endocrine 4: 91–105.
27. Quack, M., and C. Carlberg. 2000. Ligand-triggered stabilization of vitamin D receptor/retinoid X receptor heterodimer conformations on DR4-type response elements. J. Mol. Biol. 296: 743–756.
28. Ozfirat Z, Chowdhury TA. Vitamin D deficiency and type 2 diabetes. Postgrad Med J. 2010;86:18–25.
29. Kayaniyil S, Vieth R, et al. Association of vitamin D with insulin resistance and b-cell dysfunction in subjects at risk for type 2 diabetes. Diabetes Care. 2010;33(6):1379–81
30. Khoo AL, Chai LY, Koenen HJ, Oosting M, Steinmeyer A, Zuegel U, Joosten I, Netea MG, van der Ven AJ. Vitamin D(3) down-regulates proinflammatory cytokine response to Mycobacterium tuberculosis through pattern recognition receptors while inducing protective cathelicidin production. Cytokine. 2011 Aug;55(2):294-300.
31. [Viaene L](http://www.ncbi.nlm.nih.gov/pubmed?term=Viaene L%5BAuthor%5D&cauthor=true&cauthor_uid=23171504), [Evenepoel P](http://www.ncbi.nlm.nih.gov/pubmed?term=Evenepoel P%5BAuthor%5D&cauthor=true&cauthor_uid=23171504), [Meijers B](http://www.ncbi.nlm.nih.gov/pubmed?term=Meijers B%5BAuthor%5D&cauthor=true&cauthor_uid=23171504), [Vanderschueren D](http://www.ncbi.nlm.nih.gov/pubmed?term=Vanderschueren D%5BAuthor%5D&cauthor=true&cauthor_uid=23171504), [Overbergh L](http://www.ncbi.nlm.nih.gov/pubmed?term=Overbergh L%5BAuthor%5D&cauthor=true&cauthor_uid=23171504), [Mathieu C](http://www.ncbi.nlm.nih.gov/pubmed?term=Mathieu C%5BAuthor%5D&cauthor=true&cauthor_uid=23171504). Uremia Suppresses Immune Signal-Induced CYP27B1 Expression in Human Monocytes. [Am J Nephrol.](http://www.ncbi.nlm.nih.gov/pubmed?term=Uremia Suppresses Immune Signal-Induced CYP27B1 Expression in Human Monocytes) 2012;36(6):497-508
32. Stefano Sammicheli, Nicolas Ruffin, Rebecka Lantto, Nancy Vivar, Francesca Chiodi, Bence Rethi IL-7 modulates B cells survival and activation by inducing BAFF and CD70 expression in T cells Journal of Autoimmunity 000 (2012) 1e11
33. Mazzucchelli R, Durum SK. Interleukin-7 receptor expression: intelligent design. Nat Rev Immunol 2007;7:144e54.
34. Mackay F, Schneider P, Rennert R et al. BAFF and April: a tutorial on B cell survival. Annu Rev Immunol 2003; 21: 231–264
35. Nicolas Janus, Launay-Vincent Vacher, Svetlana Karie, Elena Ledneva and Gilbert Deray Vaccination and chronic kidney disease Nephrol Dial Transplant (2008) 23: 800–807
36. [Hashemi B](http://www.ncbi.nlm.nih.gov/pubmed?term="Hashemi B"%5BAuthor%5D), [Mahdavi-Mazdeh M](http://www.ncbi.nlm.nih.gov/pubmed?term="Mahdavi-Mazdeh M"%5BAuthor%5D), [Abbasi M](http://www.ncbi.nlm.nih.gov/pubmed?term="Abbasi M"%5BAuthor%5D), [Hosseini-Moghaddam SM](http://www.ncbi.nlm.nih.gov/pubmed?term="Hosseini-Moghaddam SM"%5BAuthor%5D), [Zinat NH](http://www.ncbi.nlm.nih.gov/pubmed?term="Zinat NH"%5BAuthor%5D), [Ahmadi F](http://www.ncbi.nlm.nih.gov/pubmed?term="Ahmadi F"%5BAuthor%5D). Efficacy of HBV vaccination in various stages of chronic kidney disease: is earlier better? [Hepat Mon.](http://www.ncbi.nlm.nih.gov/pubmed/22224080) 2011 Oct 1;11(10):816-20.
37. Bouts A, Davin J, Krediet R et al. Children with chronic renal failure have reduced numbers of memory B cells. Clin Exp Immunol 2004; 137: 589–594
38. Fernandez-Fresnedo G, Ramos MA, Gonzalez-Pardo MC *et al*. B lymphopenia in uremia is related to an accelerated *in vitro* apoptosis and dysregulation of Bcl-2. *Nephrol Dial Transplant* 2000; 15: 502–510
39. [Pahl MV](http://www.ncbi.nlm.nih.gov/pubmed?term=Pahl MV%5BAuthor%5D&cauthor=true&cauthor_uid=19684120), [Gollapudi S](http://www.ncbi.nlm.nih.gov/pubmed?term=Gollapudi S%5BAuthor%5D&cauthor=true&cauthor_uid=19684120), [Sepassi L](http://www.ncbi.nlm.nih.gov/pubmed?term=Sepassi L%5BAuthor%5D&cauthor=true&cauthor_uid=19684120), [Gollapudi P](http://www.ncbi.nlm.nih.gov/pubmed?term=Gollapudi P%5BAuthor%5D&cauthor=true&cauthor_uid=19684120), [Elahimehr R](http://www.ncbi.nlm.nih.gov/pubmed?term=Elahimehr R%5BAuthor%5D&cauthor=true&cauthor_uid=19684120), [Vaziri ND](http://www.ncbi.nlm.nih.gov/pubmed?term=Vaziri ND%5BAuthor%5D&cauthor=true&cauthor_uid=19684120). Effect of end-stage renal disease on B-lymphocyte subpopulations, IL-7, BAFF and BAFF receptor expression. Nephrol Dial Transplant (2010) 25: 205–212
40. [Sammicheli S](http://www.ncbi.nlm.nih.gov/pubmed?term=Sammicheli S%5BAuthor%5D&cauthor=true&cauthor_uid=22194871), [Dang VP](http://www.ncbi.nlm.nih.gov/pubmed?term=Dang VP%5BAuthor%5D&cauthor=true&cauthor_uid=22194871), [Ruffin N](http://www.ncbi.nlm.nih.gov/pubmed?term=Ruffin N%5BAuthor%5D&cauthor=true&cauthor_uid=22194871), [Pham HT](http://www.ncbi.nlm.nih.gov/pubmed?term=Pham HT%5BAuthor%5D&cauthor=true&cauthor_uid=22194871), [Lantto R](http://www.ncbi.nlm.nih.gov/pubmed?term=Lantto R%5BAuthor%5D&cauthor=true&cauthor_uid=22194871), [Vivar N](http://www.ncbi.nlm.nih.gov/pubmed?term=Vivar N%5BAuthor%5D&cauthor=true&cauthor_uid=22194871), [Chiodi F](http://www.ncbi.nlm.nih.gov/pubmed?term=Chiodi F%5BAuthor%5D&cauthor=true&cauthor_uid=22194871), [Rethi B](http://www.ncbi.nlm.nih.gov/pubmed?term=Rethi B%5BAuthor%5D&cauthor=true&cauthor_uid=22194871). IL-7 Promotes CD95-Induced Apoptosis in B Cells via the IFN-c/STAT1 Pathway Stefano. [PLoS One.](http://www.ncbi.nlm.nih.gov/pubmed?term=IL-7 Promotes CD95-Induced Apoptosis in B Cells via the IFN-c%2FSTAT1 Pathway) 2011;Dec 6(12):e28629.
41. [Baeke F](http://www.ncbi.nlm.nih.gov/pubmed?term=Baeke F%5BAuthor%5D&cauthor=true&cauthor_uid=20302932), [Korf H](http://www.ncbi.nlm.nih.gov/pubmed?term=Korf H%5BAuthor%5D&cauthor=true&cauthor_uid=20302932), [Overbergh L](http://www.ncbi.nlm.nih.gov/pubmed?term=Overbergh L%5BAuthor%5D&cauthor=true&cauthor_uid=20302932), [van Etten E](http://www.ncbi.nlm.nih.gov/pubmed?term=van Etten E%5BAuthor%5D&cauthor=true&cauthor_uid=20302932), [Verstuyf A](http://www.ncbi.nlm.nih.gov/pubmed?term=Verstuyf A%5BAuthor%5D&cauthor=true&cauthor_uid=20302932), [Gysemans C](http://www.ncbi.nlm.nih.gov/pubmed?term=Gysemans C%5BAuthor%5D&cauthor=true&cauthor_uid=20302932), [Mathieu C](http://www.ncbi.nlm.nih.gov/pubmed?term=Mathieu C%5BAuthor%5D&cauthor=true&cauthor_uid=20302932). Human T lymphocytes are direct targets of 1,25-dihydroxyvitamin D3 in the immune system. [J Steroid Biochem Mol Biol.](http://www.ncbi.nlm.nih.gov/pubmed?term=Human T lymphocytes are direct targets of 1%2C25-dihydroxyvitamin D3 in the immune system) 2010 Jul;121(1-2):221-7
42. [Booth J](http://www.ncbi.nlm.nih.gov/pubmed?term=Booth J%5BAuthor%5D&cauthor=true&cauthor_uid=20824286), [Wilson H](http://www.ncbi.nlm.nih.gov/pubmed?term=Wilson H%5BAuthor%5D&cauthor=true&cauthor_uid=20824286), [Jimbo S](http://www.ncbi.nlm.nih.gov/pubmed?term=Jimbo S%5BAuthor%5D&cauthor=true&cauthor_uid=20824286), [Mutwiri G](http://www.ncbi.nlm.nih.gov/pubmed?term=Mutwiri G%5BAuthor%5D&cauthor=true&cauthor_uid=20824286). Modulation of B cell responses by Toll-like receptors. [Cell Tissue Res.](http://www.ncbi.nlm.nih.gov/pubmed/20824286) 2011 Jan;343(1):131-4
43. Heil F. et al.,. Species-specific recognition of single-stranded RNA via toll-like receptor 7 and 8. Science. 2004: 303(5663):1526-9
44. [Hemmi H](http://www.ncbi.nlm.nih.gov/pubmed?term=Hemmi H%5BAuthor%5D&cauthor=true&cauthor_uid=11130078), [Takeuchi O](http://www.ncbi.nlm.nih.gov/pubmed?term=Takeuchi O%5BAuthor%5D&cauthor=true&cauthor_uid=11130078), [Kawai T](http://www.ncbi.nlm.nih.gov/pubmed?term=Kawai T%5BAuthor%5D&cauthor=true&cauthor_uid=11130078), [Kaisho T](http://www.ncbi.nlm.nih.gov/pubmed?term=Kaisho T%5BAuthor%5D&cauthor=true&cauthor_uid=11130078), [Sato S](http://www.ncbi.nlm.nih.gov/pubmed?term=Sato S%5BAuthor%5D&cauthor=true&cauthor_uid=11130078), [Sanjo H](http://www.ncbi.nlm.nih.gov/pubmed?term=Sanjo H%5BAuthor%5D&cauthor=true&cauthor_uid=11130078), [Matsumoto M](http://www.ncbi.nlm.nih.gov/pubmed?term=Matsumoto M%5BAuthor%5D&cauthor=true&cauthor_uid=11130078), [Hoshino K](http://www.ncbi.nlm.nih.gov/pubmed?term=Hoshino K%5BAuthor%5D&cauthor=true&cauthor_uid=11130078), [Wagner H](http://www.ncbi.nlm.nih.gov/pubmed?term=Wagner H%5BAuthor%5D&cauthor=true&cauthor_uid=11130078), [Takeda K](http://www.ncbi.nlm.nih.gov/pubmed?term=Takeda K%5BAuthor%5D&cauthor=true&cauthor_uid=11130078), [Akira S](http://www.ncbi.nlm.nih.gov/pubmed?term=Akira S%5BAuthor%5D&cauthor=true&cauthor_uid=11130078). A Toll-like receptor recognizes bacterial DNA. [Nature.](http://www.ncbi.nlm.nih.gov/pubmed?term=Hemmi%2C H. et al. (2000) Nature 408%3A740–745.) 2000 Dec 7;408(6813):740-5
45. [Kaisho T](http://www.ncbi.nlm.nih.gov/pubmed?term=Kaisho T%5BAuthor%5D&cauthor=true&cauthor_uid=11501880), [Akira S](http://www.ncbi.nlm.nih.gov/pubmed?term=Akira S%5BAuthor%5D&cauthor=true&cauthor_uid=11501880). Toll-like receptors and their signaling mechanism in innate immunity. [Acta Odontol Scand.](http://www.ncbi.nlm.nih.gov/pubmed/11501880) 2001 Jun;59(3):124-30
46. [Takeda K](http://www.ncbi.nlm.nih.gov/pubmed?term=Takeda K%5BAuthor%5D&cauthor=true&cauthor_uid=12524386), [Kaisho T](http://www.ncbi.nlm.nih.gov/pubmed?term=Kaisho T%5BAuthor%5D&cauthor=true&cauthor_uid=12524386), [Akira S](http://www.ncbi.nlm.nih.gov/pubmed?term=Akira S%5BAuthor%5D&cauthor=true&cauthor_uid=12524386). Toll-like receptors. [Annu Rev Immunol.](http://www.ncbi.nlm.nih.gov/pubmed/12524386) 2003;21:335-76.
47. [Hayashi T](http://www.ncbi.nlm.nih.gov/pubmed?term=Hayashi T%5BAuthor%5D&cauthor=true&cauthor_uid=22041420), [Nakamura T](http://www.ncbi.nlm.nih.gov/pubmed?term=Nakamura T%5BAuthor%5D&cauthor=true&cauthor_uid=22041420), [Takaoka A](http://www.ncbi.nlm.nih.gov/pubmed?term=Takaoka A%5BAuthor%5D&cauthor=true&cauthor_uid=22041420). Pattern recognition receptors. [Nihon Rinsho Meneki Gakkai Kaishi.](http://www.ncbi.nlm.nih.gov/pubmed/22041420) 2011;34(5):329-45.
48. [Jeong E](http://www.ncbi.nlm.nih.gov/pubmed?term=Jeong E%5BAuthor%5D&cauthor=true&cauthor_uid=21488180), [Lee JY](http://www.ncbi.nlm.nih.gov/pubmed?term=Lee JY%5BAuthor%5D&cauthor=true&cauthor_uid=21488180). Intrinsic and extrinsic regulation of innate immune receptors. [Yonsei Med J.](http://www.ncbi.nlm.nih.gov/pubmed/21488180) 2011 May;52(3):379-92
49. [Brown J](http://www.ncbi.nlm.nih.gov/pubmed?term=Brown J%5BAuthor%5D&cauthor=true&cauthor_uid=20940366), [Wang H](http://www.ncbi.nlm.nih.gov/pubmed?term=Wang H%5BAuthor%5D&cauthor=true&cauthor_uid=20940366), [Hajishengallis GN](http://www.ncbi.nlm.nih.gov/pubmed?term=Hajishengallis GN%5BAuthor%5D&cauthor=true&cauthor_uid=20940366), [Martin M](http://www.ncbi.nlm.nih.gov/pubmed?term=Martin M%5BAuthor%5D&cauthor=true&cauthor_uid=20940366). TLR-signaling networks: an integration of adaptor molecules, kinases, and cross-talk. [Brown J](http://www.ncbi.nlm.nih.gov/pubmed?term=Brown J%5BAuthor%5D&cauthor=true&cauthor_uid=20940366), [Wang H](http://www.ncbi.nlm.nih.gov/pubmed?term=Wang H%5BAuthor%5D&cauthor=true&cauthor_uid=20940366), [Hajishengallis GN](http://www.ncbi.nlm.nih.gov/pubmed?term=Hajishengallis GN%5BAuthor%5D&cauthor=true&cauthor_uid=20940366), [Martin M](http://www.ncbi.nlm.nih.gov/pubmed?term=Martin M%5BAuthor%5D&cauthor=true&cauthor_uid=20940366). [J Dent Res.](http://www.ncbi.nlm.nih.gov/pubmed?term=TLR-signaling Networks%3A An Integration of Adaptor Molecules%2C Kinases%2C and Cross-talk) 2011 Apr;90(4):417-27
50. [Alvarez-Rodriguez L](http://www.ncbi.nlm.nih.gov/pubmed?term=Alvarez-Rodriguez L%5BAuthor%5D&cauthor=true&cauthor_uid=22345707), [Lopez-Hoyos M](http://www.ncbi.nlm.nih.gov/pubmed?term=Lopez-Hoyos M%5BAuthor%5D&cauthor=true&cauthor_uid=22345707), [Garcia-Unzueta M](http://www.ncbi.nlm.nih.gov/pubmed?term=Garcia-Unzueta M%5BAuthor%5D&cauthor=true&cauthor_uid=22345707), [Amado JA](http://www.ncbi.nlm.nih.gov/pubmed?term=Amado JA%5BAuthor%5D&cauthor=true&cauthor_uid=22345707), [Cacho PM](http://www.ncbi.nlm.nih.gov/pubmed?term=Cacho PM%5BAuthor%5D&cauthor=true&cauthor_uid=22345707), [Martinez-Taboada VM](http://www.ncbi.nlm.nih.gov/pubmed?term=Martinez-Taboada VM%5BAuthor%5D&cauthor=true&cauthor_uid=22345707). Age and low levels of circulating vitamin D are associated with impaired innate immune function. [J Leukoc Biol.](http://www.ncbi.nlm.nih.gov/pubmed?term=Age and low levels of circulating vitamin D are associated with impaired innate immune function) 2012 May;91(5):829-38.
51. Dickie, L. J., Church, L. D., Coulthard, L. R., Mathews, R. J., Emery, P., McDermott, M. F. (2010) Vitamin D3 down-regulates intracellular Tolllike receptor 9 expression and Toll-like receptor 9-induced IL-6 production in human monocytes. *Rheumatology (Oxford)* **49,** 1466–1471


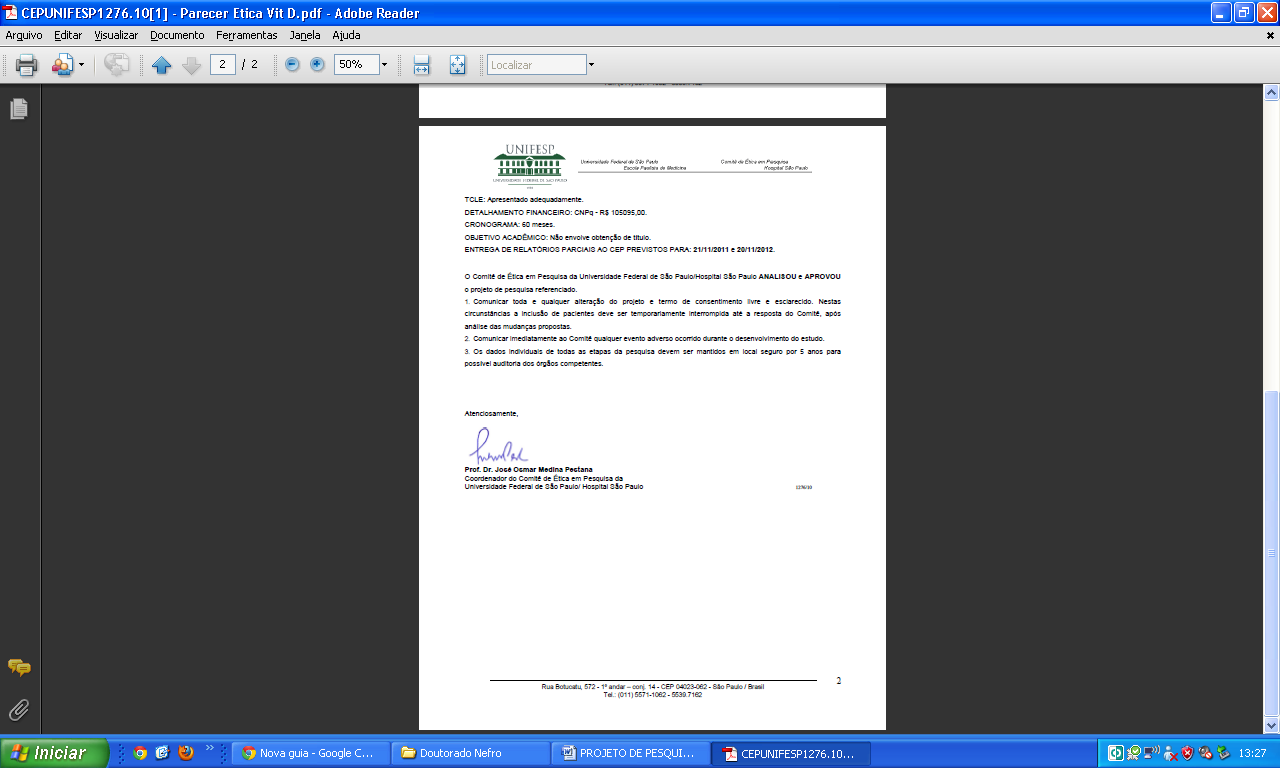

Supplement: S1 Protocol — (DOC) [file pone.0179540.s008.doc]
